# Sex-specific gut microbiome dynamics in *Labeo catla*: links to reproductive hormones, metabolic dimorphism, and environmental factors

**DOI:** 10.3389/fmicb.2025.1651975

**Published:** 2025-11-13

**Authors:** Jitendra Kumar Sundaray, Madhusmita Mohapatra, Avinash Rasal, Uday Kumar Udit, Sriprakash Mohanty, Debasrita Mohanty, Ipsita Iswari Das, Lakshman Sahoo, Pramoda Kumar Sahoo

**Affiliations:** ICAR-Central Institute of Freshwater Aquaculture, Kausalyaganga, Bhubaneswar, Odisha, India

**Keywords:** gut microbiota, high-throughput sequencing, functional diversity, environmental factors, biological factors

## Abstract

**Introduction:**

Gut microbiota play a critical role in aquaculture by enhancing nutrient metabolism, digestion, immune response, and reproductive performance in fish. *Labeo catla*, one of the most commercially important Indian major carps, demands better reproductive management; however, insights into its gut microbiome composition and functional dynamics, particularly during the crucial pre-spawning phase, remain limited.

**Methods:**

In this study, we investigated the structural and functional attributes of gut microbial communities in male and female *L. catla* reared in ICAR-CIFA ponds using high-throughput Illumina MiSeq 16S rRNA gene sequencing and examined their associations with reproductive traits.

**Results and discussion:**

Histological analysis confirmed active gametogenesis in both sexes, while hormonal assays showed higher estradiol levels in females compared to males. Microbiome profiling revealed Proteobacteria as the dominant phylum, followed by Fusobacteria, Bacteroidetes, and Firmicutes. *Cetobacterium* and *Shewanella* were the most prevalent genera, with sex-specific differences in microbial diversity and composition. Functional prediction analysis identified genes associated with reproduction, lipid metabolism, digestion, and immunity. Correlation studies revealed a negative association between *Bacteroidetes* and 11-KT, while *Shewanella* and *Serratia* showed positive correlations with estradiol, indicating a potential role of gut microbes in modulating reproductive readiness. Canonical Correspondence and variance partitioning analyses revealed that gut microbiome variation in male and female *L. catla* was predominantly influenced by biological factors (63.97%) compared to environmental factors (20.99%). Notably, despite their low abundance, *Clostridium perfringens* and *Pseudomonas stutzeri* were identified as keystone taxa significantly shaping microbial network structure and stability.

**Conclusion:**

This study provides the first comprehensive insight into the sex-specific gut microbiome's dynamics in *L. catla* during the pre-spawning season and highlight their application in broodstock management and sustainable aquaculture practices.

## 1 Introduction

Aquaculture is the fastest-growing sector within agriculture, and India ranks second globally in fisheries production (FAO, 2020). The sector plays a critical role in rural employment, livelihood generation, and nutritional security through the provision of high-quality animal protein (Sundaray et al., 2022). India is often referred to as the “carp country,” with Indian major carps (IMCs) forming the backbone of freshwater aquaculture. Among them, *Labeo catla* (Catla) is one of the most economically significant species, widely farmed across the Indian subcontinent and neighboring countries including Bangladesh, Nepal, and Myanmar (Sahoo et al., 2020, 2023; Manam and Quraishi, 2024). Catla's popularity is attributed to its rapid growth, large size, superior meat quality, and palatability, making it a preferred species for aquaculture development and livelihood support.

Sexual dimorphism in fish reflects differences in morphology, physiology, and behavior between males and females and is often regulated by underlying molecular and endocrine mechanisms (Mei and Gui, 2015). Among these, the gut microbiome has emerged as a key player influencing host metabolism, immunity, disease resistance, and even reproductive function (Wang et al., 2018; Foysal et al., 2020; Hoque et al., 2023). Gut microbial composition is shaped by both intrinsic factors such as host age, genetics, and hormonal status and extrinsic factors including diet, environmental conditions, and habitat water quality (Wong and Rawls, 2012; Clements et al., 2014; Egerton et al., 2018). Aquatic organisms, in particular, are highly responsive to abiotic factors such as pH, dissolved oxygen, nitrogen levels, and water hardness, which can significantly alter gut microbial communities (Tan et al., 2019).

Reproductive processes in fish are tightly regulated by the hypothalamic-pituitary-gonadal (HPG) axis, where hormones like follicle-stimulating hormone (FSH), luteinizing hormone (LH), 11-ketotestosterone (11-KT), and estradiol (E2) play pivotal roles in gametogenesis and gonadal maturation (Amenyogbe et al., 2020; Pan et al., 2024; Han et al., 2024). Emerging evidence suggests that gut microbes may modulate these hormonal pathways, but such interactions remain poorly understood in aquaculture species such as *L. catla*.

Previous studies on IMC gut microbiota largely relied on conventional culture-based methods (Ghosh et al., 2010; Ray et al., 2010; Khan and Ghosh, 2012; Mandal and Ghosh, 2013; Banerjee et al., 2015; Talukdar et al., 2016), which offer limited resolution. Recent advancements in high-throughput sequencing technologies, such as next-generation sequencing (NGS) and metagenomics, have enabled a more detailed and accurate analysis of microbial community structure and function (Ghanbari et al., 2015; Tarnecki et al., 2017; Gallo et al., 2020; Bhat et al., 2023). However, NGS-based studies targeting IMCs, particularly *L. catla*, remain scarce (Tyagi and Singh, 2017; Mukherjee et al., 2020; Foysal et al., 2020).

To bridge this knowledge gap, the present study aimed to explore the gut bacterial communities of male and female *L. catla* during the pre-spawning phase using high-throughput 16S rRNA gene sequencing. Specifically, we sought to: (i) assess sex-specific variations in gut microbial diversity and composition, and functional potential in relation to reproduction, growth, and immunity, (ii) examine the influence of abiotic and biotic parameters, including serum hormone levels, on gut microbial composition, and (iii) analyze bacterial co-occurrence patterns to reveal potential interspecies interactions within the gut microbiota. The pre-spawning period represents a critical physiological window characterized by heightened hormonal activity and reproductive tissue development, offering an ideal context to explore microbiome-host dynamics. Insights from this study could inform microbiome-based strategies to enhance reproductive performance and overall fish health, thereby supporting sustainable aquaculture practices.

## 2 Materials and methods

### 2.1 Study area and collection of fish and tissue samples

For the present investigation, *L. catla* fish (*n* = 10; 5 males and 5 females) were collected from the two rearing ponds of ICAR-CIFA, Bhubaneswar, India during June 2022. The fish were not subjected to vaccination and appeared to be in good health, showing no signs of infection. All fish were anesthetized using MS-222 also known as (a.k.a.) (tricaine methanesulfonate, Sigma Aldrich, USA) at a dose of 0.1 g L^−1^ and dissected immediately after catching. For microbial analysis, only foregut tissue samples were carefully excised, and separated from each fish under aseptic conditions, providing 5 biological replicates per sex. Tissue samples were collected using sterile scissors and scalpels, and stored individually at −20°C until further analyses. The blood samples were collected from each pre-weighed fish via the caudal peduncle and transferred to a microfuge tube at room temperature for 4 h to allow coagulation. The tubes were centrifuged at 1500 × *g* for 20 min and supernatant was collected to obtain serum. All experiments were approved by the Institutional Animal Ethics Committee of ICAR-CIFA, Bhubaneswar, Odisha, India. Animal studies were conducted in compliance with ARRIVE 2.0 guidelines, which include considerations for study design, sample size determination, randomization, and statistical analysis.

### 2.2 Environmental and biological parameters

Surface water samples were collected once at the time of fish sampling from two rearing ponds at ICAR-CIFA to avoid any temporal bias. Using a sterile plastic bottle, three independent water samples were collected from each pond to provide biological replicates for physico-chemical analysis. Environmental parameters, e.g., water temperature, pH, conductivity, alkalinity, hardness, dissolved oxygen (DO), phosphate (${\text{PO}}_{4}^{3-}$-P), ammonia (${\text{NH}}_{4}^{+}$-N), and nitrite (${\text{NO}}_{2}^{-}$-N) in each rearing pond were assessed following the method outlined by Leoni et al. (2018). Body weight (W_t_), standard length (SL), gonad weight (GW), liver weight (LW), and gut weight (GtW) were measured for both male and female fish. Eviscerated weight (EW) (i.e., whole fish excluding the gonad, liver, and gut tissue) was calculated. Moreover, additional bioenergetic indices were computed to evaluate the fish's condition. Gonadosomatic index (GSI) expressed as 100 × GW/EW served as a marker for fish reproductive cycles. Hepatosomatic index (HSI) was calculated as 100 × LW/EW which provided insights into liver function and metabolic health, and fatness (K) was determined as 100 × W/L^3^ offering an indicator of adiposity and overall fish condition. These indices together offer valuable insights into the reproductive dynamics as well as the physiological state of the fish species under investigation.

### 2.3 Histological analysis of gonadal sections

Gonadal tissues were fixed in 10% neutral buffered formalin for over 24 h before undergoing a dehydration process through a graded ethanol series. The samples were then cleared with xylene, embedded in paraffin, and sectioned into 4 μm slices using a microtome. If necessary, the section ribbons were floated on water, then transferred onto pre-cleaned albumenized glass slides and placed on a slide warmer at 60 °C for fixation. The slides were incubated for 12 h to ensure complete stretching. Subsequently, the paraffin sections were stained using the standard hematoxylin and eosin (H and E) protocol. This involved dewaxing in xylene, rehydrating through a graded ethanol series, staining the nuclei with hematoxylin, and counterstaining the cytoplasm with eosin. Finally, the slides were sealed with resin. Images were captured at 10 × and 40 × magnifications using a Nexscope RXLr-5 microscope equipped with a camera (Radical Procam). ImageJ software was also used for further analyses.

### 2.4 Quantification of serum steroid hormones in *L. catla*

The hormone levels of 11-KT, estradiol, FSH, and LH in the serum of *L. catla* were quantified using enzyme-linked immunosorbent assay (ELISA) kits specific for fish (GENLISA; Krishgen Biosystems, USA; Product Code- LH: KLF0040; 11-KT: KLF0000; Estradiol: KLF0245; FSH: KLF0039). The ELISA assays were conducted following the manufacturer's protocol, and absorbance measurements were recorded using an iMark™ Microplate Absorbance Reader (Bio-Rad, India). The assay procedure involved the simultaneous incubation of the target analyte with a biotin-labeled antibody, followed by washing and the addition of an affinity-labeled horseradish peroxidase (HRP) conjugate. After further incubation and washing steps to remove unbound enzyme conjugates, substrates A and B were introduced, leading to a colorimetric reaction. The intensity of the color developed was directly proportional to the concentration of the respective hormone in the sample. All standards and samples were analyzed in triplicate to ensure accuracy and reproducibility.

### 2.5 Extraction of DNA, library construction, and sequencing of 16S rRNA genes

DNA from foregut tissues (100 mg) and surface water (600 μl) samples was extracted using the DNeasy PowerSoil Pro Kit (Qiagen) following manufacturer's instructions. Extracted DNA samples were quantified with Thermo Scientific™ NanoDrop™ 2000/2000 c Spectrophotometers, USA. DNA quality and reliability were checked using 0.8% agarose gel electrophoresis, and subsequently kept at −20 °C for further processing. The samples underwent quality assessment, demonstrating satisfactory yield and concentration levels, which render them suitable for Illumina library preparation (Supplementary Table 1).

The V3-V4 hyper-variable region of the 16S rRNA gene was amplified via PCR utilizing the primer pair 341F-805R (Liu et al., 2020). The PCR protocol comprised an initial denaturing step at 95 °C for 5 min, followed by 30 cycles of denaturation at 94 °C for 30 sec, annealing at 56 °C for 30 sec, and extension at 72 °C for 30 sec. A final extension step was conducted at 72 °C for 10 min to complete the reaction. The PCR products were then migrated on an agarose gel and purified using the Qiagen^TM^ QIAquick PCR Purification Kit. Subsequently, library indexing and preparation were performed using the Nextera method. Finally, Illumina MiSeq sequencing was carried out using a 2 × 300 bp paired-end sequencing protocol. Briefly, adapter sequences were added to the primer pairs to align with Illumina index and sequencing adapters. Master mixes contained total DNA (12.5 ng) and KAPA HiFi HotStart ReadyMix (2 ×; KAPA Biosystems, Wilmington, MA). Negative controls (no template) were also included and amplified. Amplicons were purified using AMPure XP reagent. Samples were then amplified with Illumina sequencing adapters and dual-index barcodes i.e., index 1(i7) and index 2(i5) followed by purification, quantification, and normalization before pooling.

### 2.6 Sequence raw data processing and downstream analyses

Data de-multiplexing was performed initially based on the Illumina 3^rd^ index reads through bcl2fastq v.2.20 software, which facilitated the conversion of raw files to fastq files. Subsequently, sequenced data quality was assessed using FastQC v.0.11.8 (Andrews, 2017). The low-quality bases (< q30) were filtered off during read pre-processing using Trimgalore v.0.4.01 (Krueger, 2015) and used for downstream analysis. The raw sequences obtained from this study have been deposited in SRA—Sequence Read Archive (SUB14182840) under the BioProject ID: PRJNA1070889.

### 2.7 Bioinformatics analysis

The initial FASTQ files were subjected to quality filtering and subsequent merging, employing the QIIME 2 (Quantitative Insights into Microbial Ecology) software v.2018.11 (Caporaso et al., 2010). Following the quality filtering and merging procedures, which incorporated the use of DADA2 software for error correction in Illumina-sequenced amplicons, the outcomes consisted of (i) a feature table showing ASVs (i.e., Operational Taxonomic Units OTUs) and (ii) a feature data table with sequences linked with these ASVs (Callahan et al., 2016). Any potential contaminants originating from fish gut, like chloroplasts and mitochondria, were eliminated from the final files. Taxonomic assignment was performed and aligned with the Greengenes reference OTU database (Langille et al., 2013). Venn diagram was generated to detect the unique and shared OTUs by using a web-based tool i.e., Bioinformatics Tools for genomics and transcriptomics analysis (https://www.biotools.fr/misc/venny).

Diversity analysis was performed on nine rarefaction depths (ASVs rarefied at 1,667, 3,334, 5,000, 6,667, 8,333, 10,000, 11,666, 13,333, and 15,000 sequences per sample) by analyzing alpha rarefaction curves. Sequence coverage of ≥97% was supported by QIIME2 diversity plugin-generated rarefaction curves. α and β-diversity estimates were calculated using PAST software v.4.03. Several α-diversity metrics were estimated, including richness (observed species i.e., Sobs, Chao1) as well as the diversity indices (Shannon, Simpson). Spearman's correlations (ρ) were computed and identified abiotic factors that are strongly associated with richness, revealing the key environmental drivers of bacterial α-diversity. Distances of β-diversity i.e., Bray-Curtis (taxonomic) as well as UniFrac (phylogenetic) [weighted (abundance matrix), and un-weighted (presence and/or absence matrix)] distances were calculated to explore the differences among the structure of gut bacterial communities between the sample groups (Lozupone and Knight, 2005). Both Bray-Curtis and weighted UniFrac dissimilarity matrices of these bacterial groups were graphically visualized by two-dimensional NMDS (non-metric multidimensional scaling) plots. Pairwise dissimilarity assessment of β-diversity between sex-specific groups (i.e, male and female) was performed via Multivariate Analysis of Variance (MANOVA), a non-parametric test. One-way ANOVA, followed by Games-Howell *post-hoc* test (*p*-value < 0.05) was applied to compare significant differences in α-and β-diversity indices between *L. catla* gut sample groups.

The variation within the bacterial communities was evaluated by using SIMPER (Similarity Percentage Analysis) based on Bray-Curtis distance matrix. This analysis was performed in PAST v.4.03, a software application designed for statistical analysis and data visualization. To identify variations in taxa composition, LEfSe analysis, i.e., Linear Discriminant Analysis (LDA) Effect Size, was applied (Segata et al., 2011). Taxa meeting the criteria where their logarithmic base 10 of LDA score >2.0 and FDR (false discovery rate) < 0.05 determined by the Kruskal-Wallis test, were regarded as differential taxa. One-way ANOVA were performed to assess the sex stages on serum hormone levels. The Mantels' pearson correlation test was utilized to assess relationship between the bacterial community and serum hormones, conducted using the PAST v.4.03 software.

We used PICRUSt2 analysis i.e., Phylogenetic Investigation of Communities by Reconstruction of Unobserved States to anticipate bacterial functionality (Douglas et al., 2019). Metagenome prediction accuracy was estimated by calculating the NSTI value i.e., Nearest Sequenced Taxon Index. Normalization of OTU table was carried out, taking into account the expected 16S copy number for each organism, and these OTU tables were further employed to predict the functional profile using KOs (Kyoto Encyclopedia of Genes and Genomes i.e., KEGG Orthologs). To aggregate all KEGG Orthologs (KOs) into their respective KEGG pathways, the KOs were grouped by their functions. The role of individual OTU in influencing a specific gene function was determined by calculating the product of OTU abundances and the corresponding gene abundances for each taxon. We also examined functional categories within the gut microbiomes of male and female *L. catla*, comparing aspects like molecular communication, disease suppression, nutrition, and other factors crucial for growth promotion and fitness. The outcomes from PICRUSt2 were examined using a software, STAMP v.2.1.3 (Statistical Analysis of Metagenomic Profiles), employing Welch's two-sided *t*-test (i.e., for unequal variance) to detect significant variation in bacterial functional abundances between male and female sex types (Parks et al., 2014). Extended error bar plots were utilized across three KEGG pathway levels for visualization.

Canonical correspondence analysis (CCA) was carried out to explore the link between the gut bacterial community compositions of distinct sex-specific *L. catla* groups and their essential biological and environmental factors. Co-occurrence networks were created to investigate the inter-taxa relationship in the gut microbiomes specific to each sex of the *L. catla* fish. The algorithm for Network Analysis for Metagenomic Abundance Profiles calculated Pearson's correlation coefficients “*r*” using “MetagenoNets” (web-based tool available at https://web.rniapps.net/metagenonets) (Nagpal et al., 2020). Taxa were filtered using a maximum prevalence threshold of ≥0.0001, and they were included if they occurred in at least 10% of the samples. For further analysis, we exclusively considered relatively strong correlations (|*r*| > 0.7, *p*-value < 0.01) through 100 bootstrapping iterations, and the visualization of the networks was conducted using the Gephi platform (v.0.10.1) accessible at https://gephi.org. Moreover, several descriptive measures of co-occurrence networks, including network-level features (e.g., nodes, edges, average degree, clustering coefficient, graph density, modularity, and statistical inference) and node-level features (e.g., degree, closeness centrality, betweenness centrality, and eigenvector centrality) were calculated using Gephi software v.0.10.1 for each network. Average degree represents the mean number of connected edges per node. Average clustering coefficient denotes the mean ratio of node connectivity to the maximum connectivity among the connected nodes. This analysis aimed to discern variations in sex-specific *L. catla* gut microbiome co-occurrences and identify keystone species. The top 4 nodes were considered as keystone taxa, which followed high values of closeness centrality and average degree, including low scores of betweenness centrality (Berry and Widder, 2014).

## 3 Results

### 3.1 Environmental and biological factors of *L. catla* groups

Detailed information on environmental variables, e.g., water temperature, pH, conductivity, alkalinity, hardness, DO, ${\text{PO}}_{4}^{3-}$P, ${\text{NH}}_{4}^{+}$-N, and ${\text{NO}}_{2}^{-}$-N are listed in Table 1. The data on the essential biological characteristics of the fish demonstrated a consistent progression in the male fish groups, with SL, Wt, LW, EW, and HSI all showing an increasing trend. During the same period, noteworthy variations in GW and GSI were observed between the two distinct groups, with the female fish displaying higher values (Table 1).

**Table 1:** Basic environmental parameters of fish ponds and biological parameters of the male and female groups of *L*. *catla*.

**Environmental parameters**	**Pre-spawning season**	
Water temperature (°C)	30.50 ± 0.71	
Conductivity (μS cm^−1^)	0.24 ± 0.003	
pH	7.85 ± 0.07	
Alkalinity (mg L^−1^)	92.50 ± 3.54	
Hardness (mg L^−1^)	97.50 ± 3.54	
DO (mg L^−1^)	3.35 ± 0.21	
${\text{PO}}_{4}^{3}$-P (mg L^−1^)	0.01 ± 0.002	
${\text{NH}}_{4}^{+}$-N (mg L^−1^)	0.07 ± 0.02	
NO_2_-N (mg L^−1^)	0.01 ± 0.002	
**Biological parameters**	**Male**	**Female**
Body standard length (cm)	49.26 ± 1.85^a^	48.7 ± 1.48^a^
Body weight (kg)	1.90 ± 0.21^a^	1.89 ± 0.05^a^
Gonad weight (gm)	12.90 ± 1.57^a^	202.7 ± 35.04^b^
Liver weight (gm)	27.20 ± 6.30^a^	23.50 ± 3.20^a^
Gut weight (gm)	26.20 ± 1.14^a^	26.82 ± 0.61^a^
Eviscerated weight (kg)	1.84 ± 0.21^a^	1.64 ± 0.05^a^
GSI (%)	0.71 ± 0.13^a^	12.35 ± 2.22^b^
HSI (%)	1.48 ± 0.26^a^	1.43 ± 0.22^a^
K	1.59 ± 0.08^a^	1.65 ± 0.17^a^

Means with different letters indicate significant differences between two different fish groups at one-way ANOVA, *p*-value < 0.05. DO: dissolved oxygen; ${\text{NO}}_{2}^{-}$-N: nitrite, ${\text{NH}}_{4}^{+}$-N: ammonia, ${\text{PO}}_{4}^{3-}$-P: phosphate; GSI, gonadosomatic index; HSI, hepatosomatic index; K, fatness.

### 3.2 Histological analysis of male and female gonads of *L. catla*

**Figure 1:**
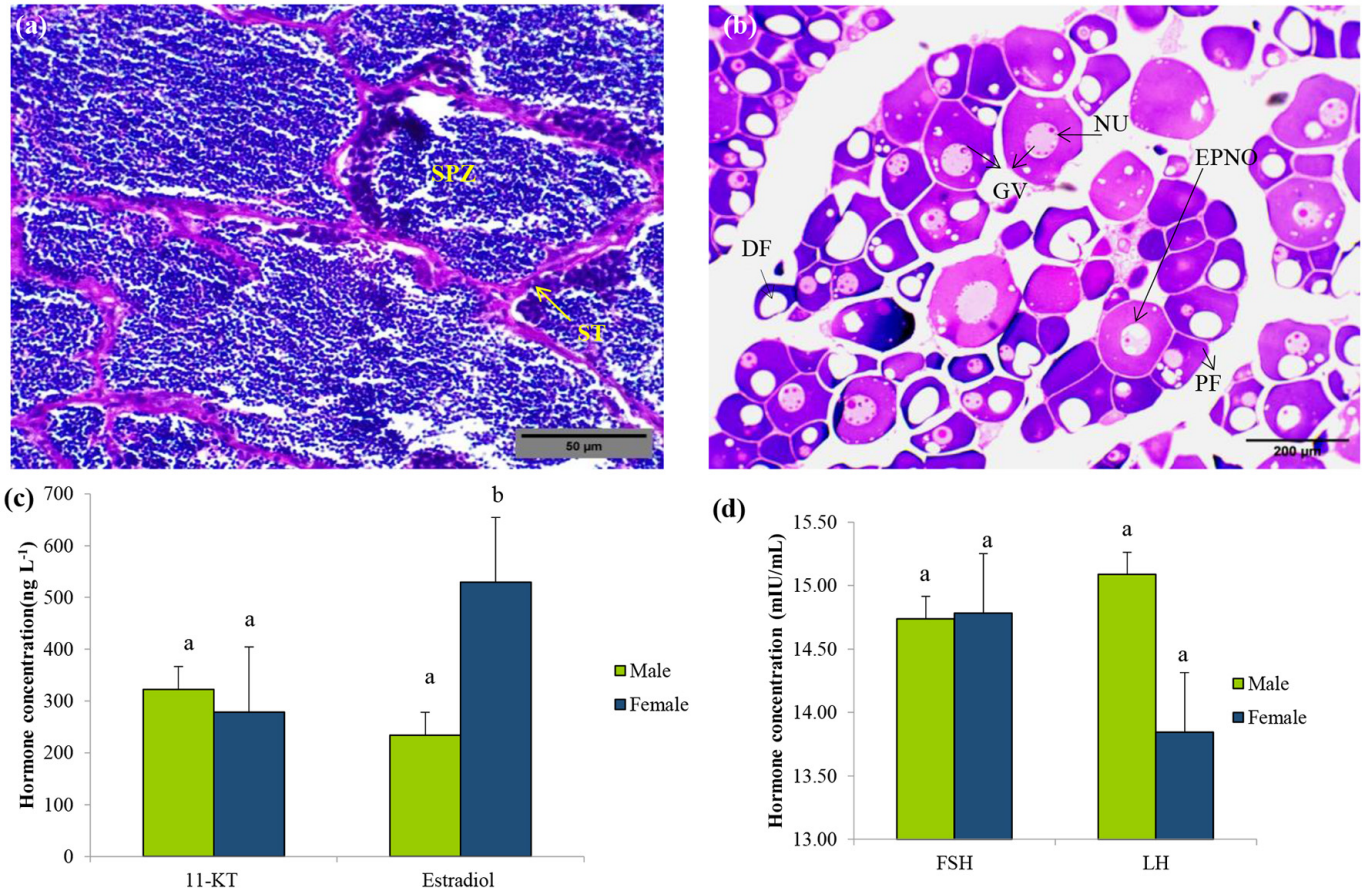
Sex identification of *L. catla* during the pre-spawning reproductive cycle. Representative histology graphs from testis **(a)** and ovary **(b)** of *L. catla* with the scale bar 500 μm and 200 μm, respectively. Serum concentrations of 11-Ketotestosterone (11-KT) and estradiol **(c)**, follicle-stimulating hormone (FSH), and luteinizing hormone (LH) **(d)** in male and female *L. catla*. Values are mean ± SEM. Different letters represent a significant difference at one-way Anova, *p*-value < 0.05. SPZ, spermatazoa; ST, Seminiferous tubules; DF, Developing follicle; NU, nucleolus; GV, germinal vesicle; PF, previtellogenic follicles; EPNO, early perinucleolar oocyte.

Histological analysis of gonadal tissues revealed clear morphological features indicative of active gametogenesis in both male and female specimens (Figures 1A, B). In the testicular sections, the presence of spermatogonial cells along the basal lamina and primary spermatocytes beneath it signifies the initiation of spermatogenesis. The testes exhibited a well-developed tubular structure, with intertubular connective tissue sparsely populated by sustentacular cells (Figure 1A). Although a few spermatozoa were observed in certain areas of the testes, they appeared to be in an immature stage, suggesting ongoing development.

Ovarian sections displayed oocytes at various stages of development, including early perinucleolar, cortical alveolar, and pre-vitellogenic stages (Figure 1B). The ovarian tissue was characterized by Stage I differentiation, comprising primary follicles and cells in the cortical alveolus stage. Follicular cells were observed surrounding the ovarian follicles, indicating their role in supporting oocyte maturation. The presence of asynchronously developing oocytes suggests a batch-spawning reproductive strategy. These observations confirm active gametogenic processes in both sexes and provide valuable insights into the reproductive status and maturation stages of the analyzed specimens.

### 3.3 Serum hormone level analysis

Serum hormone levels (11-KT, estradiol, FSH, and LH) of *L. catla* were measured during pre-spawning stages (Figures 1C, D). No significant differences in 11-KT, FSH, and LH levels were observed between male and female (*p*-value > 0.05). However, one-way ANOVA revealed significant differences in estradiol levels between the sexes (*p*-value < 0.05).

### 3.4 Sequencing results and quality control

For bacterial communities, data from the 16S rRNA gene sequencing run produced 2,640,687 reads for 10 samples from both female (*n* = 5) and male (*n* = 5) fish. After quality filtering and removal of the chloroplast, mitochondria, and other non-target ASVs, 2,384,007 reads were retained (Supplementary Table 1) representing 1,194 OTUs from gut samples. Individual samples had a wide range of reads (minimum = 75,349; maximum = 643,407; mean = 238,401 ± 192,955). Full sequencing data are publicly available at NCBI BioProject ID PRJNA1070889.

### 3.5 Bacterial community composition of *L. catla* gut

**Figure 2:**
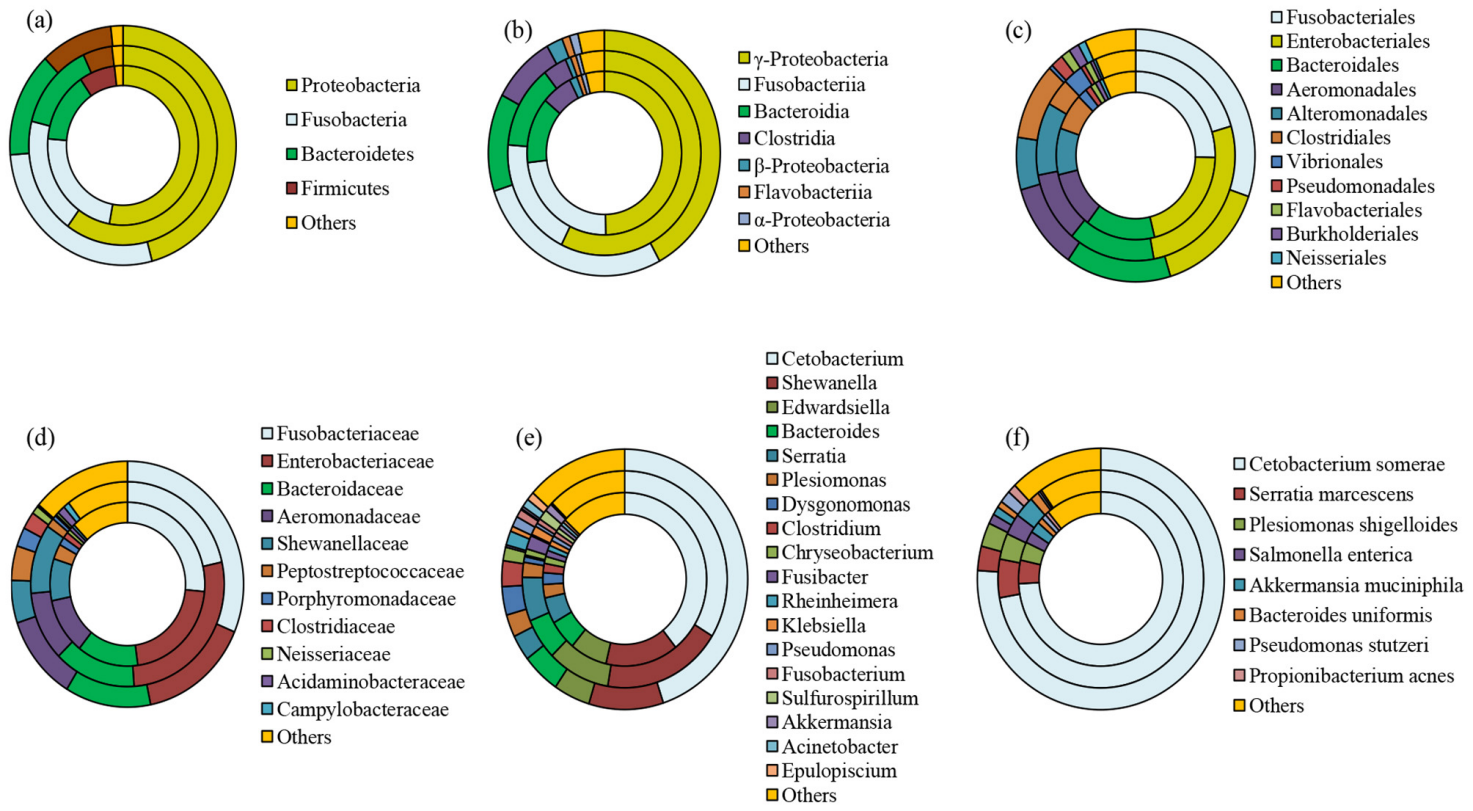
Taxonomic composition of whole, male, and female bacterial communities at phylum **(a)**, class **(b)**, order **(c)**, family **(d)**, genus **(e)**, and species **(f)** level obtained from *L. catla* gut samples. The category “others” accounted for taxa that were represented by < 1% abundance. Innermost circle represents the bacterial composition of entire catla gut (CGtotal); Middle circle: female catla gut (CGF); Outer circle: male catla gut (CGM).

MiSeq Illumina sequencing of female *L. catla* gut tissue samples (*n* = 5) resulted in a total of 647,044 reads. After quality filtering, each sample had a mean of 129,408.8 ± 56,437.5 sequences. A total of 35 bacterial phyla were identified that were mainly belonged to Proteobacteria (59.97%), Fusobacteria (18.93%), Bacteriodetes (14.20%), and Firmicutes (5.02%), together accounting for over 97% of total bacterial communities (Figure 2A). In the female gut samples, the dominant classes were γ-Proteobacteria (57.32%), Fusobacteriia (18.97%), Bacteroidia (13.15%), and Clostridia (3.97%) (Figure 2B). At the order level, female *L. catla* gut bacterial communities were greatly signified by Enterobacteriales (26.57%), Fusobacteriales (20.50%), Bacteroidales (14.04%), Alteromonadales (11.18%), Aeromonadale (10.90%), Clostridiales (4.29%), Vibrionales (3.27%), and Flavobacteriales (1.03%) (Figure 2C). Within the 218 different families, the most predominant taxa collectively represent over 85% of the total number of sequences were Enterobacteriaceae (27.37%), Fusobacteriaceae (21.54%), Bacteroidaceae (13.71%), Shewanellaceae (10.98%), Aeromonadaceae (10.96%), Peptostreptococcaceae (1.47%), Acidaminobacteraceae (1.30%), and Campylobacteraceae (1.00%) (Figure 2D). Of the 400 definite genera, *Cetobacterium* (33.76%), *Shewanella* (18.91%), *Edwardsiella* (9.92%), *Serratia* (6.56%), *Bacteroides* (6.16%), *Plesiomonas* (2.22%), *Fusibacter* (2.04%), *Sulfurospirillum* (1.57%), *Klebsiella* (1.33%), and *Akkermansia* (1.19%) were the most dominant (Figure 2E).

Sequencing of male *L. catla* gut tissue samples (*n* = 5) produced a total of 1,736,963 sequences, with a mean of 347,392.6 ± 225,583.2 sequences per sample. Among 50 different phyla that were detected in the male *L. catla* gut, the dominant ones were Proteobacteria (45.85%), Fusobacteria (27.86%), Bacteroidetes (14.17%), and Firmicutes (10.34%) (Figure 2A). In the male gut samples, the dominant classes were γ-Proteobacteria (42.10%), Fusobacteriia (27.88%), Bacteroidia (12.85%), Clostridia (8.97%), β-Proteobacteria (2.26%), α-Proteobacteria (1.19%) and Flavobacteriia (1.09%) (Figure 2B). At the order level, out of the 197 definite orders, Fusobacteriales (30.27%), Enterobacteriales (15.02%), Bacteroidales (14.29), Clostridiales (9.84%), Alteromonadales (6.70%), Pseudomonadales (1.69%), Burkholderiales (1.7%), Flavobacteriales (1.27%), Neisseriales (1.03%), collectively signified 92.55% of total bacterial communities in the male *L. catla* gut tissue (Figure 2C). Within the 289 different families, the most dominant taxa accounting for >85% of the total number of sequences were Fusobacteriaceae (31.36%), Enterobacteriaceae (15.45%), Bacteroidaceae (11.94%), Aeromonadaceae (11.17%), Shewanellaceae (5.60%), Peptostreptococcaceae (4.54%), Porphyromonadaceae (2.50%), Clostridiaceae (2.28%), and Neisseriaceae (1.06%) (Figure 2D). Major genera detected in male *L. catla* gut were *Cetobacterium* (44.92%), *Shewanella* (9.80%), *Bacteroides* (5.05%), *Edwardsiella* (4.80%), *Dysgonomonas* (3.53%), *Serratia* (3.21%), *Clostridium* (3.20%) *Plesiomonas* (2.79%), *Rheinheimera* (1.77%), *Chryseobacterium* (1.69%), *Pseudomonas* (1.29%), *Acinetobacter* (1.06%), *Epulopiscium* (1.05%), and *Fusobacterium* (1.02%) (Figure 2E).

Venn diagram is applied to demonstrate the shared and unique OTUs in two sex-specific *L. catla* gut bacterial groups. A total of 798 and 1,103 OTUs were observed in the female and male gut microbial groups, respectively (Supplementary Figure 1). A total of 707 OTUs were shared between the male and female guts, accounting for 59.21% in the gut microbial community. The female gut microbial group possessed the fewest unique OTUs (91; 7.62%), whereas the male gut microbial group displayed the highest number of unique OTUs (396; 33.17%).

### 3.6 Analysis of diversity of the gut microbiome of *L. catla*

#### 3.6.1 Rarefaction curve and sampling depth

**Figure 3:**
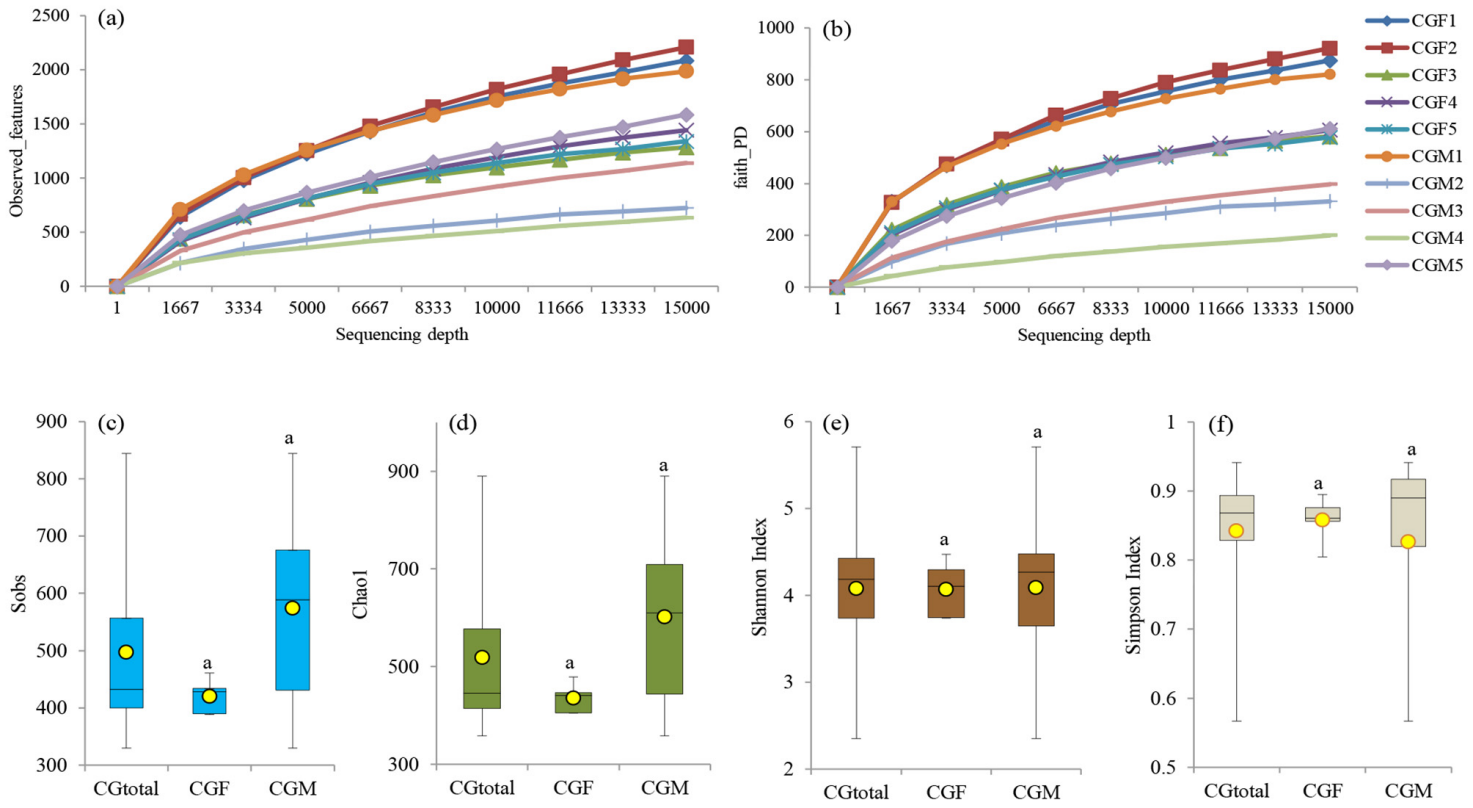
Rarefaction plots illustrating observed features **(a)** and Faith's PD **(b)** at different sequencing depths for individual samples. α-diversity metrics, such as sobs **(c)**, chao1 **(d)**, shannon **(e)**, and simpson **(f)**, are depicted for the entire gut microbiome (CGtotal) as well as the female (CGF) and male (CGM) groups of *L. catla*. Means with the same letter indicate non-significant differences (ANOVA, *p*-value > 0.05).

**Figure 4:**
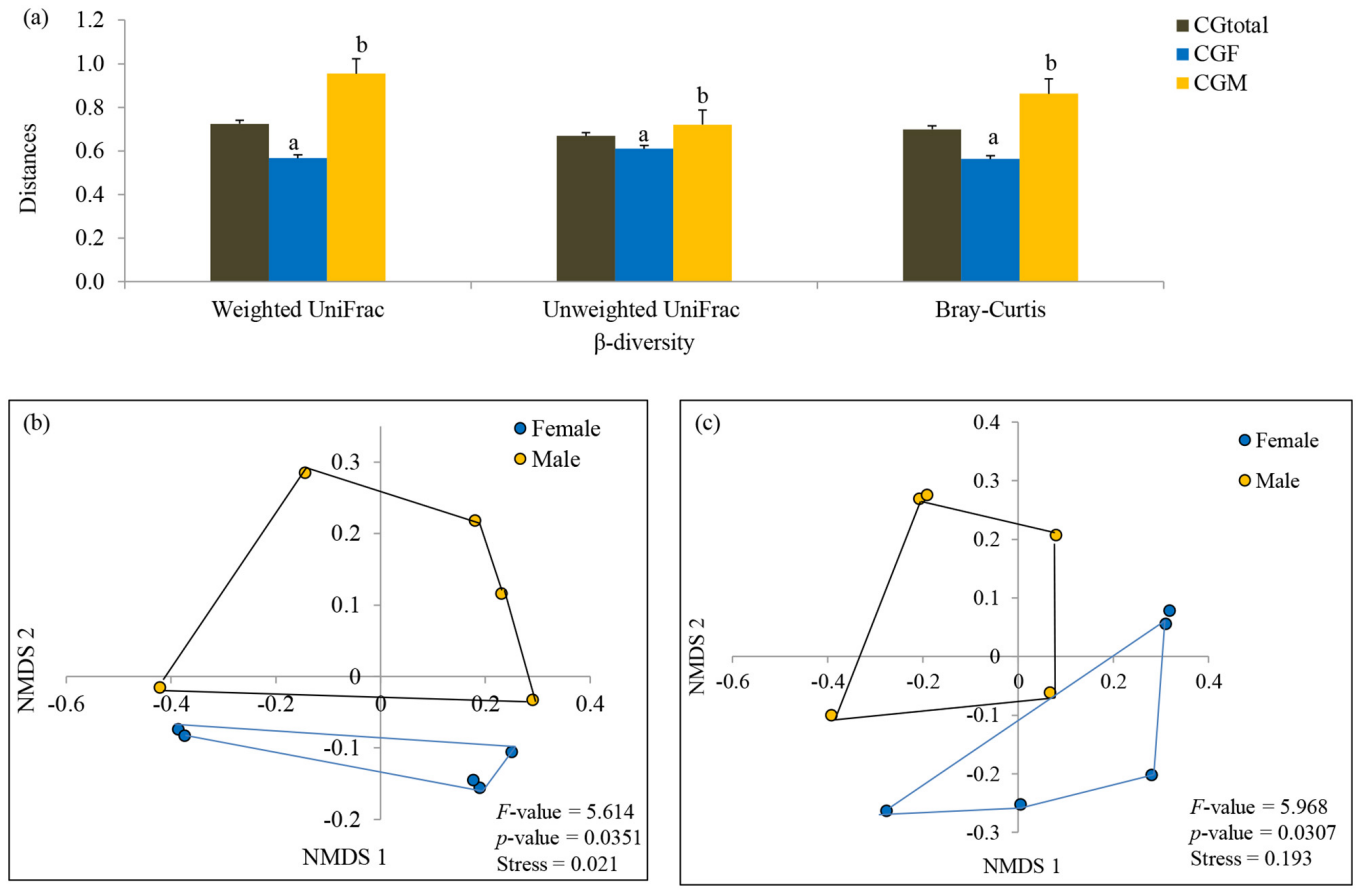
Comparison of phylogenetic (weighted and unweighted UniFrac distances) and taxonomic (Bray-Curtis distances) β-diversity of male and female *L. catla* gut taxa **(a)**. Comparison of gut microbial communities between male and female *L. catla* using NMDS based on weighted UniFrac distances **(b)** and Bray-Curtis distances **(c)**. Multivariate analysis of variance (MANOVA) was used to test significant differences in these two distinct sex-specific groups using PAST (v.3.18) program.

The rarefaction curve analysis aimed to establish the adequacy of recovered OTUs using Illumina MiSeq sequencing. The gut bacterial samples associated with *L. catla* were rarefied to 15,000 sequences to maintain a consistent sampling depth. α-rarefaction e.g., observed OTUs/features and Faith's PD i.e., phylogenetic diversity curves at different sampling depths are shown in Figures 3A, B. The rarefaction curve analysis demonstrated a high sequencing depth of the 16S rRNA gene in both male and female *L. catla* gut samples, as shown in Figures 3A, B. Saturation was observed in all the gut samples, indicating that around 15,000 sequences were sufficient to capture the full spectrum of gut bacterial diversity. These findings also signify that female *L. catla* gut bacterial samples, particularly CGF1 and CGF2, exhibit a greater diversity of species (Figures 3A, B). Subsequently, the rarefied feature table was employed to assess both α- and β-diversity (Figures 3C–F, 4A).

#### 3.6.2 α- and β-diversity indices of *L. catla* gut microbiome

α-diversity indices (e.g., Sobs, Chao1, Shannon, and Simpson) were examined to explore the richness and diversity of the gut microbiome in *L. catla*. Median and average α-diversity values of all the α-diversity parameters were lower in the female catla gut microbial samples and higher in the male gut samples, but their distinctions were not statistically significant at *p*-value > 0.05 (Figures 3C–F). Additionally, Chao1 richness index of gut bacterial communities exhibited significant Spearman's rho correlation (*p*-value < 0.01) with hardness (ρ = 0.675), ${\text{NH}}_{4}^{+}$-N (ρ = −0.679). Body weight of *L. catla* was significantly correlated with the Sobs (ρ = −0.842), Chao1 (ρ = −0.830).

β-diversity exhibited a parallel trend with significantly (one-way ANOVA, *p*-value < 0.05) higher estimates in male gut taxa [(β_Bray − Curtis_ = 0.863 vs 0.564), (β_weightedUniFrac_ = 0.954 vs 0.567), and (β_unweightedUniFrac_ = 0.720 vs 0.610)] than that of female gut taxa (Figure 4A). The β-diversity patterns in the gut taxa of male and female *L. catla* were examined via NMDS, utilizing Bray-Curtis and weighted UniFrac distances (Figures 4B, C). A distinct clustering of gut samples was observed using both the Bray-Curtis and UniFrac distances, delineating male and female groups. This bacterial community composition varied significantly (MANOVA, *p*-value < 0.001) between these two groups. NMDS analysis also demonstrated a high degree of variability within each sex-specific bacterial community (Figures 4B, C).

Through SIMPER analysis, the investigation of taxa exhibiting the most substantial variations in relative abundance between two distinct sex-specific groups was conducted. The findings emphasized that families such as Fusobacteriaceae, Enterobacteriaceae, Bacteroidaceae, Shewanellaceae, Aeromonadaceae, Peptostreptococcaceae, Porphyromonadaceae, Clostridiaceae, Acidaminobacteraceae, Weeksellaceae, Chromatiaceae and Campylobacteraceae were the primary contributors (>80%) to the observed structural distinctions in gut microbial communities between males and females (Table 2). Within the genus, *Cetobacterium, Shewanella, Edwardsiella, Serratia, Bacteroides, Dysgonomonas, Clostridium*, etc. differed significantly by 53.25% between male and female catla guts.

**Table 2:** SIMPER analysis showing taxa that contributed to the difference between male and female *L. catla* gut bacterial communities at family (a) and genus (b) level.

**Contribution of discriminating taxa**	**Average abundance**		**Average dissimilarity**	**Contribution (%)**	**Cumulative (%)**
	**Female gut**	**Male gut**			
**(a) Family level (Overall average dissimilarity: 47.18)**					
*Fusobacteriaceae*	21.50	31.40	11.27	23.89	23.89
*Enterobacteriaceae*	27.40	15.40	7.59	16.08	39.97
*Bacteroidaceae*	13.70	11.90	5.44	11.54	51.51
*Shewanellaceae*	11.00	5.60	5.11	10.83	62.34
*Aeromonadaceae*	11.00	11.20	3.99	8.46	70.80
*Peptostreptococcaceae*	1.47	4.54	1.79	3.78	74.58
*Porphyromonadaceae*	0.62	2.50	1.40	2.96	77.54
*Clostridiaceae*	0.23	2.28	1.03	2.18	79.72
*Acidaminobacteraceae*	1.30	0.18	0.64	1.36	81.08
*Weeksellaceae*	0.76	1.00	0.58	1.24	82.32
*Chromatiaceae*	0.15	0.99	0.50	1.07	83.38
*Campylobacteraceae*	1.00	0.10	0.49	1.04	84.42
**(b) Genus level (Overall average dissimilarity: 53.25)**					
*Cetobacterium*	33.80	44.90	14.97	28.11	28.11
*Shewanella*	18.90	9.80	8.82	16.57	44.68
*Edwardsiella*	9.92	4.80	5.19	9.75	54.43
*Serratia*	6.56	3.21	2.56	4.81	59.24
*Bacteroides*	6.16	5.05	2.38	4.47	63.71
*Dysgonomonas*	0.84	3.53	2.00	3.76	67.47
*Clostridium*	0.25	3.20	1.49	2.80	70.27
*Plesiomonas*	2.22	2.79	1.24	2.33	72.61
*Fusibacter*	2.04	0.29	1.01	1.89	74.50
*Chryseobacterium*	0.93	1.69	0.95	1.79	76.29
*Rheinheimera*	0.22	1.77	0.90	1.69	77.98
*Sulfurospirillum*	1.57	0.15	0.76	1.43	79.41
*Akkermansia*	1.19	0.31	0.57	1.07	80.48

### 3.7 Differential abundances in the male and female *L. catla* gut microbiome

LEfSe analysis was carried out to exemplify and highlight significant variations in the gut bacterial abundances among distinct sex-specific *L. catla* groups (Supplementary Figure 2). The analysis revealed that two families viz. Clostridiaceae, Exiguobacteraceae, and one genus (*Aquitalea*) were significantly different and higher (LDA score > 2.0; *p*-value < 0.05) in male gut samples compared with the female groups. In contrast, the female gut bacterial group demonstrated markedly greater abundances of ε*-*Proteobacteria (and their sister lineages, namely, Campylobacterales, and Campylobacteraceae) compared with the male gut microbiome groups (Supplementary Figure 2). Clostridiaceae and Exiguobacteraceae exhibited a significant increase of 9.97 and 8.54 folds, respectively, in male gut samples as compared to female gut samples. Meanwhile, Campylobacteraceae showed a significant 10.54-fold increase in the female gut group compared to the male gut.

### 3.8 Predicted function of gut bacterial communities of *L. catla*

**Figure 5:**
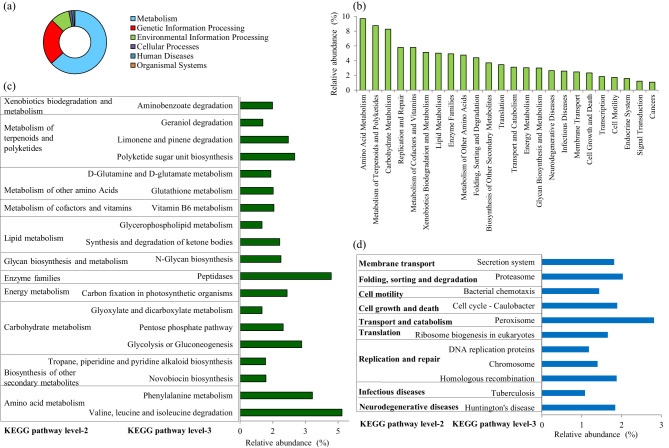
Distribution of the overall predicted function of *L. catla* gut bacterial communities. Dominant KEGG categories at level 1 **(a)**, level 2 **(b)**, and level 3 of metabolic pathways **(c)**, and level 3 of pathways within the human diseases, genetic information processing, cellular processes, and pathways **(d)** present in the gut bacterial communities of *L. catla*.

The functional profiling of bacterial communities harboring the *L. catla* intestine in male and female groups was predicted using PICRUSt assignment through 16S rRNA gene sequences (Figure 5). NSTI value i.e., 0.14 ± 0.04, determined from PICRUSt analysis, signifies high prediction accuracy. The study involved analyzing bacterial 16S rRNA gene sequencing dataset from gut samples of *L. catla* to predict genes and their functional pathways using KEGG databases. In total, 7,305 KO groups were identified, encompassing 247 pathways in *L. catla* gut samples (*n* = 10). The analysis showed that the majority (63.31%) of the detected genes at KEGG level-1 were enriched in metabolic pathways, followed by genetic information processing (23.74%), environmental information processing (9.78%), cellular processes (1.50%), human diseases (1.07%), and organismal systems (0.57%) (Figure 5A).

In addition, amino acid metabolism (9.69%), metabolism of terpenoids and polyketides (8.75%), carbohydrate metabolism (8.28%), replication and repair (5.79%), metabolism of cofactors and vitamins (5.78%), xenobiotics biodegradation and metabolism (5.11%), lipid metabolism (5.02%), enzyme families (4.92%), metabolism of other amino acids (4.77%), folding, sorting and degradation (4.38%), biosynthesis of other secondary metabolites (3.69%), translation (3.45%), transport and catabolism (3.10%), energy metabolism (3.02%), and others were detected as the major predicted functional pathways in the KEGG level-2 (Figure 5B).

The study found that at KEGG level-3, the relative abundances of genes linked with various metabolic pathways were particularly prominent (Figure 5C). These pathways included valine, leucine, and isoleucine degradation, peptidases, phenylalanine metabolism, glycolysis/gluconeogenesis, polyketide sugar unit biosynthesis, limonene and pinene degradation, carbon fixation in photosynthetic organisms, pentose phosphate pathway, N-glycan biosynthesis, synthesis and degradation of ketone bodies, vitamin B6 metabolism, glutathione metabolism, aminobenzoate degradation, D-glutamine and D-glutamate metabolism, novobiocin biosynthesis, tropane, piperidine, and pyridine alkaloid biosynthesis, geraniol degradation, glycerophospholipid metabolism, glyoxylate and dicarboxylate metabolism. Moreover, this study revealed a dominance of relative gene abundances in various pathways, including those related to genetic information processing (such as proteasome, ribosome biogenesis in eukaryotes, homologous recombination, chromosome, and DNA replication proteins), environmental information processing (secretion system), cellular processes (peroxisome, cell cycle in Caulobacter, bacterial chemotaxis), as well as genes associated with human diseases (Huntington's disease and tuberculosis) (Figure 5D).

#### 3.9.1. Metabolic pathways linked to reproduction, growth and development, immunity

Genes related to sex hormone synthesis and reproduction such as *whiEVI, CYP* (aromatase), *cypD*_E and *CYP102A2*_3 (cytochrome P450/NADPH-cytochrome P450 reductase), *CYP81F* (indol-3-yl-methylglucosinolate hydroxylase), *CYP152A* (fatty-acid peroxygenase), *CYP125A* (cholest-4-en-3-one 26-monooxygenase), *CYP142* (cholest-4-en-3-one 26-monooxygenase), *linC, CYP111A* (linalool 8-monooxygenase) were identified from both male and female *L. catla* gut samples. Genes encoding for digestive enzymes such as *bglX, bglB* (beta-glucosidase), *amyA, malS* (alpha-amylase), *treS* (maltose alpha-D-glucosyltransferase/alpha-amylase), *CBH2, cbhA* (cellulose 1,4-beta-cellobiosidase), endoglucanase, chitinase, putative chitinase, *chiA* (bifunctional chitinase/lysozyme), *chbP* (N,N'-diacetylchitobiose phosphorylase), *HEXA*_B (hexosaminidase), *nagZ* (beta-N-acetyl hexosaminidase), *ydgD* (protease), *prc* and *ctpA* (carboxyl-terminal processing protease), *lip, TGL2* (triacylglycerol lipase), *phytase, appA* (4-phytase/acid phosphatase) were also detected in *L. catla* gut. Other metabolism-related enzymes e.g., *glsA* (glutaminase) and *lipV* (lipase), and immune-related enzymes e.g., *SOD1, SOD2* (superoxide dismutase) of *L. catla* were identified.

#### 3.9.2. Metabolic pathways linked to fatty acid metabolism and biosynthesis

Lipid and fatty acid metabolism pathways related to glycerolipid metabolism, arachidonic acid metabolism, and α-linolenic acids were detected in *L. catla* gut. Genes encoding enzymes for fatty acid metabolism such as *GPD1* (glycerol-3-phosphate dehydrogenase), *FAD6, desA* (acyl-lipid omega-6 desaturase i.e., delta-12 desaturase), *PHYH* (phytanoyl-CoA hydroxylase), *IMPA, suhB* (myo-inositol-1(or 4)-monophosphatase), *ACSL, fadD* (long-chain acyl-CoA synthetase) were identified.

#### 3.9.3. Differential predictive functional profiling of gut bacterial communities in L. catla

**Figure 6:**
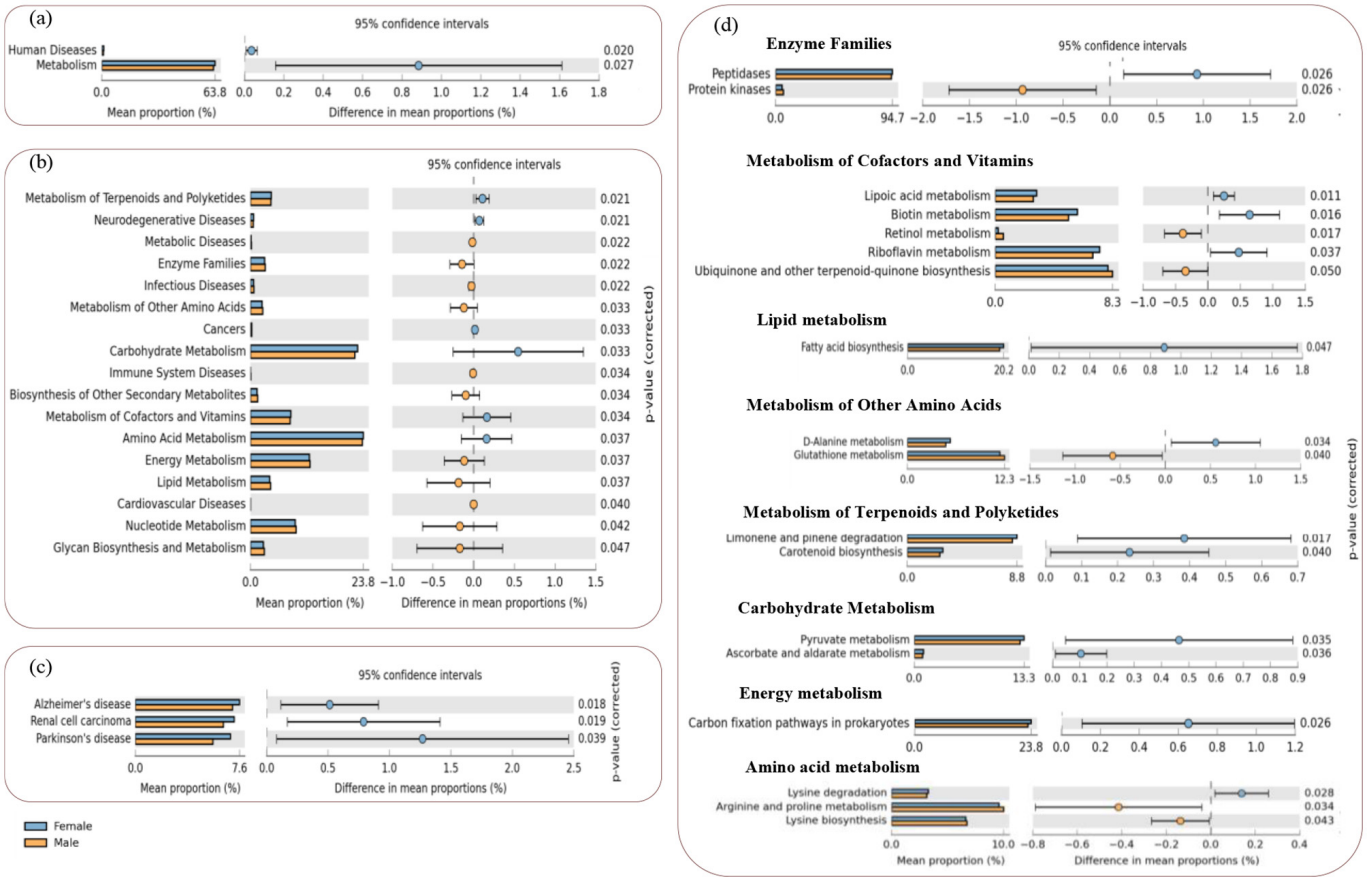
STAMP plots of PICRUSt to infer differential functions of the *L. catla* male and female gut bacterial communities (*n* = 10). KEGG Level 1 includes all pathways **(a)**, Level 2 includes metabolic and human disease related pathways **(b)**, and Level-3 includes all the significant features within the human disease **(c)** and metabolic pathways **(d)**. Significant *p*-values < 0.05 were generated by Welch's t-test.

Differential comparative analysis of KEGG functional pathways within the gut microbiome between the two groups was performed, revealing significant differences. Specifically, there were a total of 2, 15, and 14 markedly different KEGG pathways at levels 1, 2, and 3, respectively (Figure 6). Among these level-3 pathways, nine pathways were highly enriched in the female gut bacterial group, and five were enriched in the male gut bacterial group.

The expression of various bacterial functional genes linked to the KEGG metabolic pathways at level-2 (e.g., terpenoids and polyketides, carbohydrate, cofactors and vitamins, and amino acid metabolism), and human diseases (neurodegenerative and cancers) were significantly increased (*p*-value < 0.05, (Figures 6A, B) in the female *L. catla* gut bacterial groups. However, in the male *L. catla* gut bacterial groups, a markedly elevated (*p*-value < 0.05) expression of several genes linked with metabolic pathways (e.g., metabolism of energy, lipid, nucleotide, glycan biosynthesis, biosynthesis of other secondary metabolites, other amino acids, and enzyme families), as well as human diseases (e.g., cardiovascular, immune system, infectious, and metabolic diseases) were investigated (Figure 6B).

When considering the level-3 classification of KEGG metabolic pathways in the female gut bacterial group, a larger proportion of sequences were associated with lysine degradation, carbon fixation pathways in prokaryotes, pyruvate, ascorbate and aldarate metabolism, limonene and pinene degradation, carotenoid biosynthesis, fatty acid biosynthesis, D-alanine, riboflavin, biotin, and lipoic acid metabolism, peptidases (Figure 6D). While regarding the male gut bacterial group, processes related to lysine biosynthesis, arginine and proline, glutathione, retinol metabolism, ubiquinone and other terpenoid-quinone biosynthesis, and protein kinases at the level-3 classification of KEGG pathways were significantly enhanced (Figure 6D), In addition, as for the female gut bacterial groups, the expression of disease-related functional genes (e.g., Alzheimer's, Parkinson's disease, and renal cell carcinoma) were significantly high (*p*-value < 0.05, Figure 6C).

### 3.10 Relationships between bacterial community and serum hormone level

The heatmap analysis revealed significant correlations (*p*-value < 0.05) between specific bacterial phyla and genera with serum hormone levels. At the phylum level, *Bacteroidetes* showed a strong negative correlation with 11-KT (*r* = −0.756, *p*-value < 0.05) (Supplementary Figure 3A). At the genus level, *Dysgonomonas* (*r* = −0.799) and *Sulfurospirillum* (*r* = −0.729) displayed significant negative correlations (*p*-value < 0.05) with LH, and *Bacteroides* showed negative correlation with 11-KT (*r* = −0.843, *p*-value < 0.05) (Supplementary Figure 3B). In contrast, estradiol showed significant (*p*-value < 0.05) positive correlation with *Shewanella* (*r* = 0.830) and *Serratia* (*r* = 0.837). These findings suggest that specific microbial taxa may modulate reproductive hormone dynamics in *L. catla*.

### 3.11 Influence of physicochemical and biological conditions on *L. catla* gut bacterial community dynamics

**Figure 7:**
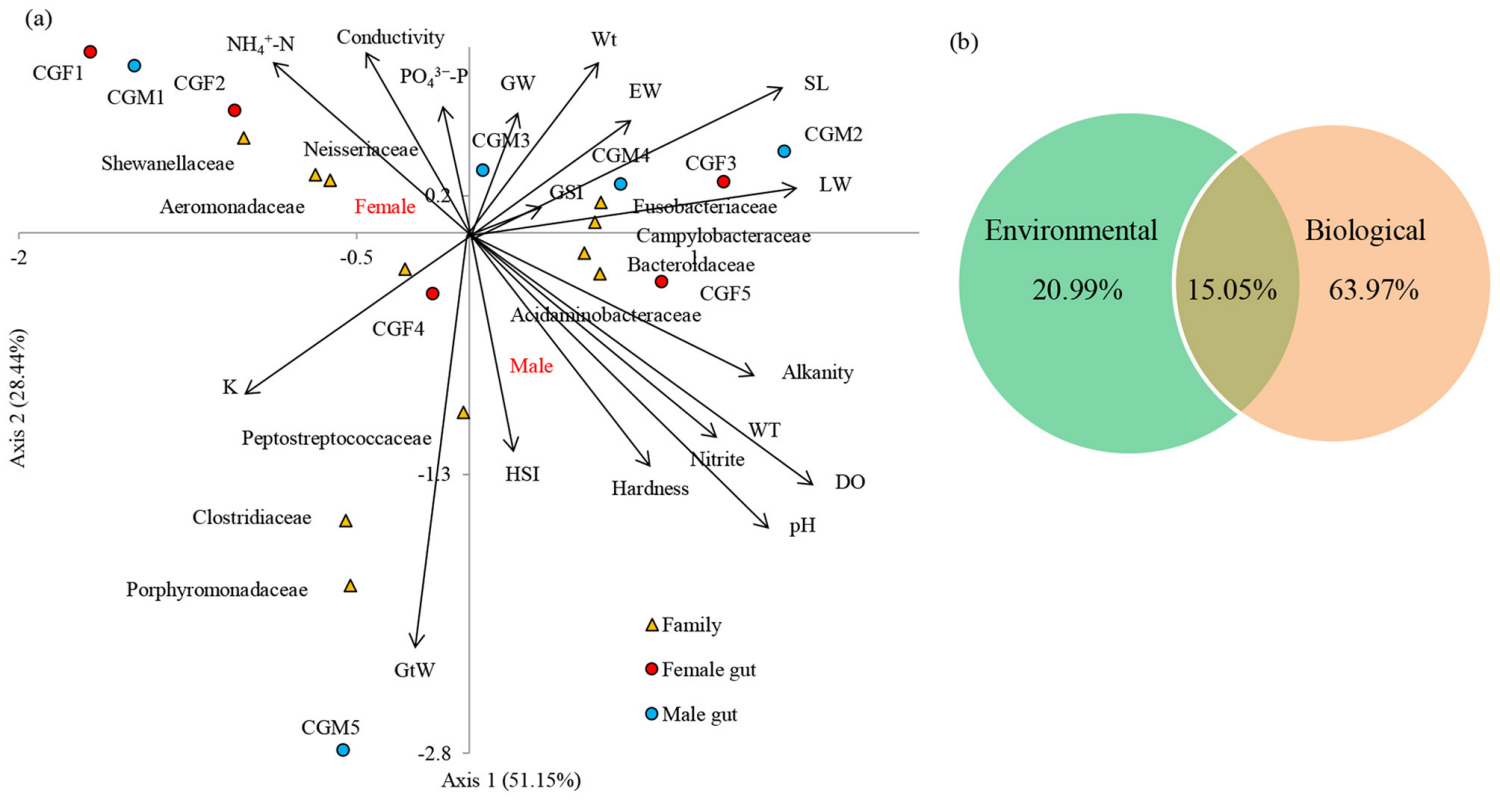
Canonical correspondence analysis (CCA) demonstrates the correlation between the bacterial compositions of two different male and female guts and their basic environmental and biological parameters at the family level **(a)**. The arrow direction of the vectors denotes the maximum change direction for a variable, and the length of each vector serves as a measure of how well the variable elucidates the data distribution. Positive correlation between the variables is depicted by long arrows in the same direction, negative correlation by arrows in opposite directions, and no correlation by perpendicular arrows. The perpendicular arrows are indicative of no correlation. VPA provided the relative contribution of environmental and biological factors in causing the variation in the *L. catla* gut bacterial taxa **(b)**. SL, standard length; W_t_, Body weight; GW, gonad weight; LW, liver weight; GtW, gut weight; EW, Eviscerated weight; GSI, gonadosomatic index; HIS, hepatosomatic index; K, fatness; WT, water temperature; ${\text{NO}}_{2}^{-}$N, nitrite-nitrogen, ${\text{NH}}_{4}^{+}$-N, ammonia-nitrogen, ${\text{PO}}_{4}^{3-}$P, dissolved orthophosphate; DO, dissolved oxygen.

Male and female *L. catla* gut microbiomes were studied to unveil the abiotic and biotic factors shaping their bacterial community structure (Figure 7A). At the order level, the cumulative variation explained by the first two CCA axes was 79.59%. The CCA axis 2 showed a strong positive association with three environmental factors such as conductivity (ρ = 0.871), ${\text{PO}}_{4}^{3}$–P (ρ = 0.741), ${\text{NH}}_{4}^{+}$-N (ρ = 0.647), whereas negatively associated with water temperature (ρ = −0.870), pH (ρ = −0.870), alkalinity (ρ = −0.869), hardness (ρ = −0.846), DO (ρ = −0.838), NO_2_–N (ρ = −0.771).

Significant correlations have been observed between gut bacterial communities and environmental factors (Supplementary Figure 4A). For instance, bacterial families namely, *Porphyromonadaceae* (*r* = 0.784 with NO_2_, *r* = 0.690 with DO, *r* = 0.672 with pH, *r* = 0.661 with WT and alkalinity, *r* = 0.649 with hardness, *r* = −0.637 with conductivity), *Bacteroidaceae* (*r* = −0.679 with NH_4_), *Campylobacteraceae* (*r* = 0.685 with NO_2_, *r* = 0.672 with DO), *Aeromonadaceae* (*r* = −0.663 with DO) and *Acidaminobacteraceae* (*r* = 0.663 with DO, *r* = 0.643 with NO_2_) were markedly (*p*-value < 0.05) associated with environmental factors of *L. catla* fish gut. Significant correlations have been observed between gut bacterial communities and the biological factors (Supplementary Figure 4B). For instance, *Enterobacteriaceae* (*r* = −0.766 with SL, *r* = 0.754 with GtW), *Porphyromonadaceae* (*r* = −0.721 with Wt, *r* = 0.681 with GtW, *r* = −0.721 with EW), *Clostridiaceae* (*r* = −0.778 with GW, *r* = −0.745 with GSI), *Acidaminobacteraceae* (*r* = −0.770 with EW, *r* = 0.685 with GSI, *r* = 0.650 with GtW), *Campylobacteraceae* (*r* = −0.779 with EW, *r* = 0.656 with GSI, *r* = 0.652 with GtW) were markedly (*p*-value 0.05) linked with biological factors of *L. catla* fish gut.

CCA based VPA i.e., variance partitioning analysis revealed that differences in the bacterial communities were mainly explained by biological (63.97%) than environmental (20.99%) factors (Figure 7B). VPA also showed that weight parameters (Wt, GW, LW, GtW, and EW) accounted for 37.34%, GSI, HIS, and K accounted for 29.04%, and body length (SL) accounted for 4.22% variance in the gut microbiome composition. The analysis also revealed that Wt accounted for the maximum variance (16.27%), followed by GtW (14.10%), K (12.70%), GSI (10.02%), EW (7.35%), GW (6.21%), LW (6.04%), and HSI (5.20%).

### 3.12 Co-presence, co-exclusion, and key taxa involved in the *L. catla* gut microbiota

**Figure 8:**
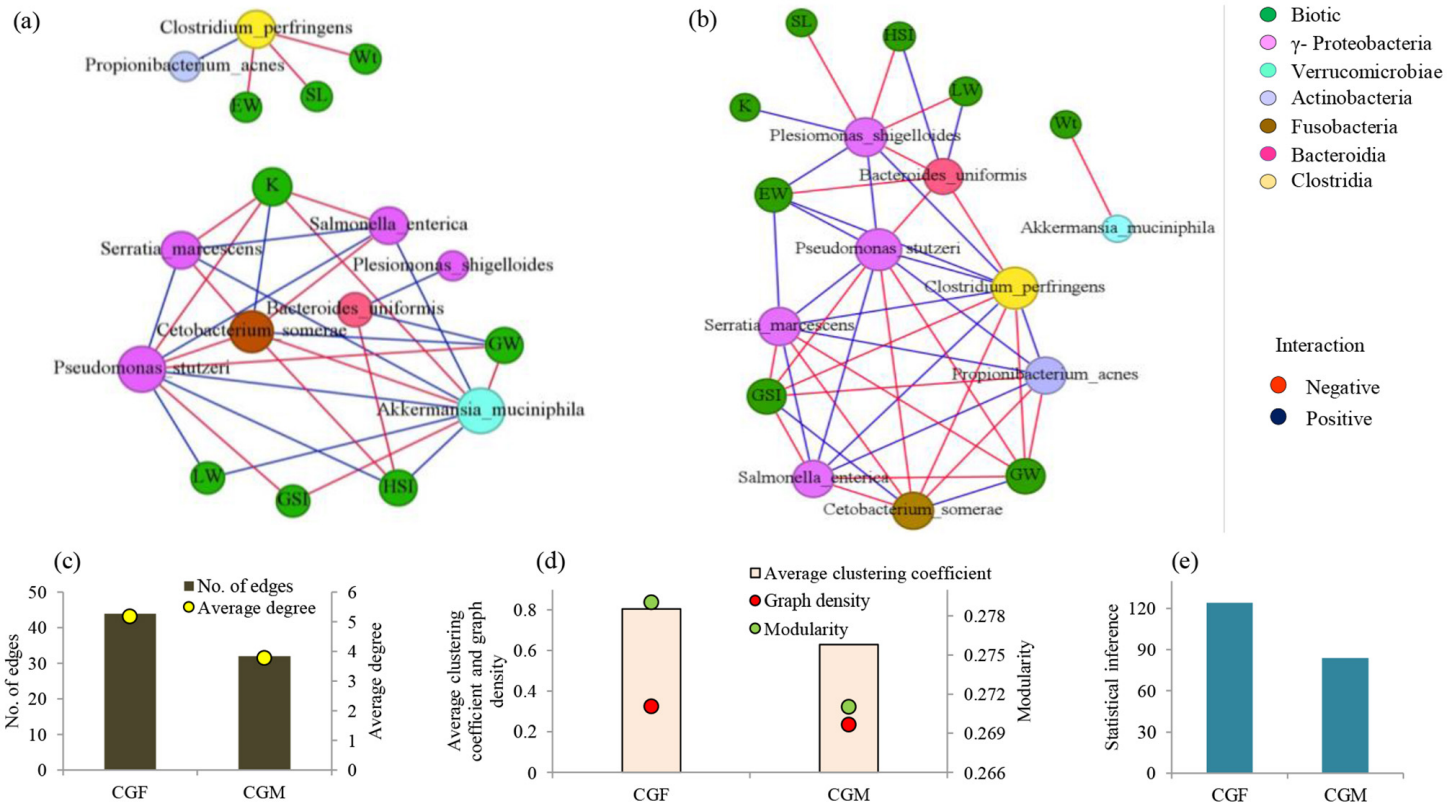
Co-occurrence networks of abundant bacterial species (>1% relative abundance) in *L. catla* gut: male **(a)** and female **(b)** groups in relation to biotic factors. Node size is proportional to species connectivity (degree), with different colors representing bacterial classes and biotic variables. Edges represent a significant correlation between two species (Pearson's |*r*| > 0.7, *p*-value < 0.01), with red showing negative and blue showing positive interactions. Network topological features of gut bacterial communities of *L. catla*, such as edges, average degree **(c)**, average clustering coefficient, graph density, modularity **(d)**, and statistical inference **(e)**. W_t_, Body weight; SL, standard length; GW, gonad weight; LW, liver weight; GtW, gut weight; EW, Eviscerated weight; GSI, gonadosomatic index; HSI, hepatosomatic index; K, fatness; CGF, female gut bacterial group; CGM, male gut bacterial group.

Co-occurrence network analysis in each *L. catla* gut bacteria group unveiled notable differences in their interactive patterns. These variances were evident in established positive and negative correlations depicted in the network plots (Figure 8). For instance, co-occurrence network indicating potential interactions among the bacterial taxa of the male gut microbiome comprised 17 nodes and 32 edges (Figure 8A). Female gut bacterial communities demonstrated a greater inter-connectedness (average clustering coefficient 0.805 vs 0.630), average degree (5.176 vs 3.765), edges (44 vs 32), graph density (0.324 vs 0.235), modularity (0.279 vs 0.271), and statistical inference (124.182 vs 83.921) as compared to the male gut bacterial communities (Figures 8C–E). Both male and female gut bacterial communities showed a maximum prevalence of negative association and the numbers of negatively correlated nodes were lower in female *L. catla* gut (52.27%) than in male fish (53.12%).

Co-occurrence network analyses emphasized the noteworthy inter-taxa interactions in *L. catla* gut bacterial communities (Figure 8). Species of the class γ-Proteobacteria were found to have strong positive associations within the gut bacterial communities of males and females. For example, *Pseudomonas stutzeri* showed strong positive correlations with *Salmonella enterica* and *Serratia marcescens*. *Salmonella enterica* was also positively correlated with *Serratia marcescens*. Furthermore, various nodes on the networks exhibited negative correlations with other nodes. For instance, *Cetobacterium somerae* (*Fusobacteriia*) showed a negative correlation with *Pseudomonas stutzeri, Salmonella enterica*, and *Serratia marcescens*.

The co-occurrence network revealed specific bacterial relationships unique to female *L. catla* gut communities, absent in males. In the female network, *Clostridium perfringens* and *Propionibacterium acnes* had strong positive relationships with *Pseudomonas stutzeri, Salmonella enterica*, and *Serratia marcescens* (Figure 8B). *Plesiomonas shigelloides* displayed significant positive correlations with *Pseudomonas stutzeri*, but these associations were absent in males (Figure 8A). Similarly, specific bacterial relationships unique to male *L. catla* gut communities were absent in females. For instance, *Akkermansia muciniphila* (Verrucomicrobiae) displayed a negative association with *Cetobacterium somerae* (Fusobacteriia) and a positive association with *Pseudomonas stutzeri, Salmonella enterica*, and *Serratia marcescens* belonging to the class γ-Proteobacteria (Figure 8B). The key taxa were represented by *Clostridium perfringens, Pseudomonas stutzeri, Plesiomonas shigelloides*, and *Serratia marcescens* (Supplementary Table 3).

## 4 Discussion

This study provides novel insights into the sex-specific differences in gut microbial communities of *L. catla* and their interplay with reproductive physiology, metabolic pathways, and environmental conditions. By integrating microbial, hormonal, and environmental analyses, we demonstrate that the gut microbiome does not merely reflect the host's physiology but may actively contribute to sex-dependent differences in reproductive readiness and metabolic adaptations. Understanding gut bacterial communities in carps is crucial for improving fish health and productivity (Wu et al., 2012; Llewellyn et al., 2014; Mukherjee et al., 2020). Our findings advance current knowledge by emphasizing the microbiome's role in IMCs, particularly in *L. catla* and its relationship with aquaculture environments, thereby offering valuable insights for better and sustainable management practices. Elevated estradiol levels in females supported active vitellogenesis and oocyte maturation, which is consistent with patterns reported in other cyprinids. In contrast, 11-KT, FSH, and LH did not differ significantly between sexes, suggesting that males and females may rely on shared endocrine mechanisms for reproductive initiation, but females exhibit unique estradiol-driven modulation of gametogenesis. Such sex-specific hormonal differences provide an important physiological backdrop to interpret variations in microbial composition and functions.

### 4.1. Gonadal histology and hormonal dynamics varied in sex-specific *L. catla*

Histological observations confirmed active gametogenesis in both male and female *L. catla*, highlighting distinct reproductive maturity during the pre-spawning phase. Testes displayed multiple spermatogenic stages, indicating continuous sperm production, while ovaries contained vitellogenic oocytes with yolk deposition, reflecting advanced reproductive readiness and batch-spawning behavior (Uribe et al., 2014; Udit et al., 2024). Ovarian histology further demonstrates that optimal dietary conditions and specific supplements, such as balanced protein-to-carbohydrate ratios and choline chloride, enhance oocyte maturation and reproductive performance, while imbalances or environmental contaminants can disrupt normal gonadal development and hormone profiles (Das et al., 2023; Udit et al., 2024). Elevated estradiol levels in females are consistently linked to active vitellogenesis and oocyte maturation, supporting findings in other cyprinids, while 11-KT, FSH, and LH levels do not show significant sex differences, suggesting shared endocrine mechanisms for reproductive initiation but a unique estradiol-driven pathway in females (Ganguly et al., 2023; Udit et al., 2024). Such sex-specific hormonal differences provide an important physiological backdrop to interpret variations in microbial composition and functions. These findings provide valuable insights into the species' reproductive status, informing effective aquaculture breeding strategies.

### 4.2 Female and male gut taxa differed in their structure, function, and diversity

The gut microbiome was dominated by members of Proteobacteria, Fusobacteria, Bacteriodetes, and Firmicutes, which are core taxa commonly observed in freshwater fish such as common carps, grass carp, as well as in the snakehead murrel (Han et al., 2010; Wu et al., 2012; Ye et al., 2014; Li H. et al., 2015; Li T. et al., 2015; Rasal et al., 2022). Notably, Proteobacteria are involved in the fish metabolism and cycling of biogeochemicals such as carbon, nitrogen, and sulfur (Fjellheim et al., 2010; Han et al., 2010). However, striking sex-based differences were detected. Within the phylum Proteobacteria, γ-Proteobacteria dominated in both male and female *L. catla* gut samples, with genera such as *Plesiomonas* (Enterobacteriaceae) and *Acinetobacter* (Moraxellaceae) commonly observed in several carp species (Namba et al., 2007; van Kessel et al., 2011). Female gut samples showed higher abundances of *Serratia marcescens, Plesiomonas shigelloides*, and *Salmonella enterica* in some samples such as CGF1 (15.26%), CGF2 (8.39%), and CGF3 (7.92%), respectively. These bacterial species can thrive as facultative pathogens in both fish and humans without causing overt disease (Walczak et al., 2017). *Serratia* generates serrawettin (a wetting agent) reducing environmental surface tension (Chan et al., 2013), while *P. shigelloides* has been linked to pathogenicity in freshwater carp (Behera et al., 2018). The natural intestinal flora exhibited stress-induced symptoms such as body darkening, hemorrhaging, fin rot, ascitic fluid accumulation, and internal organ lesions (Liu et al., 2015). *P. stutzeri* strains improved water quality, growth, gut microbiota, immune response, and hold probiotic potential for aquaculture through water supplementation (Fu et al., 2021).

Fusobacteria, particularly *Cetobacterium*, dominated male gut samples (97.51% in CGM2) and are known for butyrate and acetate production, supporting gut health, anti-inflammatory effects, and vitamin B12 synthesis (Tsuchiya et al., 2008; van Kessel et al., 2011; Wang et al., 2021). *Shewanella* was one of the abundant genera observed in both sexes of gut samples of *L. catla* in this study, produces omega-3 PUFAs i.e., polyunsaturated fatty acids (EPA: eicosapentaenoic acid and DHA: docosahexaenoic acid) and is being explored as a probiotic to enhance growth and immunity (Monroig et al., 2013; Egerton et al., 2018; Ghosh et al., 2021). Within the Firmicutes, *Clostridium* (gram-negative and obligate anaerobes) contributed to polysaccharide degradation, pathogen inhibition, SCFA production, and overall gut health, including fostering anti-inflammatory and antibacterial properties (Flint et al., 2008; Ikeda-Ohtsubo et al., 2018; Piazzon et al., 2020). In addition, our study detected the presence of *Klebsiella* aligns with previous reports of fish gut as a reservoir for opportunistic pathogens (Austin et al., 2007; Wu et al., 2012; Li H. et al., 2015; Mukherjee et al., 2020). Bacteroidetes, particularly *Bacteroides* were notably abundant in female *L. catla* gut samples, aiding in polysaccharide degradation, SCFA production, fatty acid metabolism, and overall gut function (Hu et al., 2018; Mohapatra et al., 2020; Foysal et al., 2020; Mukherjee et al., 2020). The genus also facilitates vitamin synthesis, oxygen depletion to maintain anaerobiosis, and utilization of dietary polysaccharides (Xia et al., 2014; Schwalm and Groisman, 2017), strengthening their candidacy as next-generation probiotics (Wexler and Goodman, 2017).

Sex-specific differences were evident for bacterial community composition, with female gut samples showing higher proportions of ε-Proteobacteria with a higher proportion of ε-Proteobacteria (and their sister lineages, namely, Campylobacteraceae). In contrast, male gut samples exhibited greater abundance of Clostridiaceae, Exiguobacteraceae, and Aquitalea. Members of Campylobacteraceae, often linked with reduced growth and disease symptoms in fish (Lastovica et al., 2014; Legrand et al., 2018), were more abundant in females, suggesting a correlation with lower microbial diversity (Legrand et al., 2018; Mehta et al., 2021). The genus *Exiguobacterium*, enriched in male gut samples, is recognized for its adaptability and roles in lipid droplet formation, stress tolerance, enzyme production, and amino acid release (Semova et al., 2012; Lei et al., 2014; Kasana and Pandey, 2018). The strain contributes to environmental pollutants degradation, pesticides removal, and bioremediation of arsenic, thereby mitigating oxidative stress and enhancing fish health (Shanthakumar et al., 2015; Pandey and Bhatt, 2015).

### 4.3 Functional implications of sex-specific bacterial variation in *L. catla*

Functional predictions revealed distinct metabolic signatures between sexes. In females, microbial pathways related to fatty acid biosynthesis and carbohydrate metabolism were enriched, potentially reflecting the heightened energy requirements for vitellogenesis and gonadal maturation. In contrast, males displayed enrichment of amino acid biosynthesis pathways, such as lysine metabolism, along with antioxidant pathways including glutathione metabolism. These functions may enhance protein turnover and stress tolerance, traits that are beneficial for growth and spermatogenesis. The alignment of microbial functions with sex-specific reproductive and metabolic demands underscores the adaptive significance of gut microbiota in shaping host physiology.

Lysine, an essential amino acid synthesized via the DAP, i.e., diaminopimelic acid, and AAA, i.e., aminoadipic acid, pathways (Liu et al., 2010), was significantly enriched in male microbiota. Bacterially derived lysine has been reported to contribute to host amino acid pools (Metges, 2000), suggesting that enhanced microbial lysine biosynthesis may support male growth and spermatogenesis. Likewise, glutathione-related pathways, including *gst, gpx*, and *gsr*, were more abundant in males, potentially enhancing oxidative stress tolerance, cellular detoxification, and immune regulation (Wu et al., 2004; Vallejos-Vidal et al., 2020). These sex-specific functional enrichments highlight the adaptive alignment of microbial metabolism with host reproductive and physiological needs. In both sexes, enrichment of amino acid degradation pathways, particularly for Val, Leu, and lle, was associated with fatty acid metabolism (Zhang et al., 2019), indicating a broader microbial role in nutrient turnover and metabolic health. Such microbial contributions to amino acid and lipid metabolism align with observations in trout hepatocytes (Chang et al., 2011; Dai et al., 2015), underscoring their importance for aquaculture productivity. Moreover, cellulolytic and hydrolytic enzymes (e.g., cellulase, chitinase, amylase) from gut bacteria remain vital for digesting plant-based feed ingredients, supporting host nutrient acquisition (Jiang et al., 2011; Ray et al., 2012; Ni et al., 2014).

The α and β-diversity indices are pivotal for evaluating microbiota structure (Luo et al., 2022), and in this study, diversity analysis revealed lower gut bacterial diversity in female *L. catla* compared to males, likely due to a significant water shift experienced by the female *L. catla* community during cultivation, leading to a decline in pond diversity, consistent with earlier observations in rainbow trout and Atlantic cod (Bakke et al., 2013; Wong et al., 2013). NMDS analysis further highlighted clear sex-specific differences in microbial composition, suggesting that distinct physiological demands between the sexes may potentially influence nutrient absorption, immune function, and overall health.

### 4.4 Serum hormone levels and reproductive physiology regulated by *L. catla* gut microbes

Gut microbiome showed significant correlations with serum hormone levels, suggesting potential microbial influences on reproductive endocrinology. Negative correlation between Bacteroidetes and 11-KT, as well as between *Dysgonomonas* and *Sulfurospirillum* with LH, implies that these bacterial taxa may play inhibitory roles in androgenic and luteinizing hormone regulation (Thackray, 2019). In contrast, positive correlations of *Shewanella* and *Serratia* with estradiol point toward a role in supporting vitellogenesis in females. This observation consistent with other vertebrates showing gut microbial modulation of steroidogenesis by influencing hormone metabolism and signaling pathways (Neuman et al., 2015; Colldén et al., 2019), highlight the complexity of host–microbiome–hormone interactions and underscore the need for further research to explore microbiome-targeted strategies for improving fish reproductive performance in aquaculture.

### 4.5 Abiotic factors drive gut bacterial community structure

Abiotic factors strongly influenced gut microbiota composition in *L. catla*, as indicated by CCA, which explained 79.6% of the variation. Nutrient-rich conditions with higher conductivity, phosphate, and ammonium favored bacteria involved in nitrogen and phosphorus cycling, while stable, oxygenated environments appeared less conducive for certain taxa. These results suggest that gut bacteria adapt to fluctuating water quality, with parameters such as temperature, pH, and DO shaping microbial metabolism and immune-related functions (Li et al., 2017; Zhao et al., 2018). Such insights highlight the critical role of environmental conditions in modulating fish gut microbiota and emphasize the importance of maintaining optimal water quality in aquaculture to enhance nutrient absorption, immunity, and reproductive performance.

### 4.6. Network associations between biotic parameters and key *L. catla* gut bacterial communities

Co-occurrence network analysis highlighted sex-specific variation in the *L. catla* gut microbiota interactions. Female networks exhibited higher clustering and modularity, suggesting stronger and more structured microbial associations compared to males. Network topology reflects gut bacteria interactions, and the average degree, betweenness centrality, and closeness centrality indicate the individual bacteria species' importance in influencing interactions within the network plot (Ma et al., 2016). Bacterial taxa with elevated values of betweenness centrality, positioned near the network core, likely exert significant influence on community interactions compared to other species (Li et al., 2021). Taxa within γ-Proteobacteria showed robust positive relationships and central roles in both sexes, while cross-class interactions were largely negative, reflecting competition or niche exclusion. Overall, these patterns reveal that sex influences microbial interrelationships, with positive correlations likely reflecting mutualistic adaptations to resource use and stress, while negative ones arise from competition and co-exclusion mechanisms. Notably, Verrucomicrobiae exhibited strong negative correlations with Fusobacteriia, while positive correlations were observed with γ-Proteobacteria, indicating commensalism, mutualism, symbiosis, and cooperative interactions (Coyte and Rakoff-Nahoum, 2019; Wang et al., 2024). *Akkermansia muciniphila* (Verrucomicrobiae), a mucin-degrading anaerobe, produces butyric acid that fuels gut epithelial cells and supports barrier integrity. Beyond this, it contributes to intestinal immunity and regulates glucose and lipid metabolism (Dao et al., 2015; Naito et al., 2018). Overall, these patterns reveal that sex influences microbial interrelationships, with positive correlations likely reflecting mutualistic adaptations to resource use and stress, while negative ones arise from competition and co-exclusion mechanisms (e.g., niche exclusion, direct competition for limited resources, edaphic/environmental modification, and toxin production) (Faust and Raes, 2012).

## 5 Conclusion

This study presents the first comprehensive characterization of the taxonomic composition and functional potential of gut microbiota in male and female *L. catla*, highlighting microbial dimorphism associated with sex during the pre-spawning phase. Distinct sex-specific microbial profiles were observed, with notable enrichment of species such as *Cetobacterium somerae, Serratia marcescens, Plesiomonas shigelloides, Salmonella enterica, Akkermansia muciniphila*, and *Bacteroides uniformis*. Functional predictions revealed differences in key metabolic pathways, including lipid and carbohydrate metabolism, amino acid biosynthesis, and energy production, indicating the gut mi Proteobacteria dominated microbiota's potential influence on host reproduction, growth, and immunity. Histological and hormonal analyses confirmed active gametogenesis and significantly higher estradiol levels in females, suggesting sex-specific endocrine regulation. Correlation and network analyses further highlighted the association between gut microbes and reproductive hormones, with greater microbial connectivity and stronger biological trait associations observed in females. These findings underscore the microbiome's potential role in modulating the reproductive endocrine axis and contribute valuable genomic data for future research on host–microbe interactions. Although the study is limited by the predictive nature of PICRUSt, it lays the groundwork for advanced multi-omics approaches to uncover microbial functions with higher resolution. Overall, this foundational work provides critical insights to inform microbiome-based strategies for improving reproductive performance and health in aquaculture.

## Data Availability

The datasets presented in this study have been deposited in SRA or Sequence Read Archive under the BioProject ID: PRJNA1070889.
