# Supplementary material for: Sex-specific gut microbiome dynamics in Labeo catla: links to reproductive hormones, metabolic dimorphism, and environmental factors
Source: Front Microbiol. 2025 Nov 13;16:1651975. doi: 10.3389/fmicb.2025.1651975 (PMC12657359; doi:10.3389/fmicb.2025.1651975)
Supplement: Supplementary file 1 [file Data_Sheet_1.docx]

**Appendix A: Supplementary Material**

**Sex-Specific Gut Microbiome Dynamics in *Labeo catla*: Links to Reproductive Hormones, Metabolic Dimorphism, and Environmental Factors**

Jitendra Kumar Sundaray^1*†^, Madhusmita Mohapatra^1†^, Avinash Rasal^1^, Uday Kumar Udit^1^, Sriprakash Mohanty^1^, Debasrita Mohanty^1^, Ipsita Iswari Das^1^, Lakshman Sahoo^1^, Pramoda Kumar Sahoo^1^

^1^Fish Genetics and Biotechnology Division, ICAR-Central Institute of Freshwater Aquaculture, Kausalyaganga, Bhubaneswar 751002, Odisha, India

^†^ Contributed equally

Jitendra Kumar Sundaray^1*†^, Madhusmita Mohapatra^1†^: These authors contributed equally to this work and share first authorship

***Corresponding author**:

Dr. Jitendra Kumar Sundaray

Head of Division

Fish Genetics and Biotechnology Division,

ICAR-Central Institute of Freshwater Aquaculture,

Kausalyaganga, Bhubaneswar-751002, Odisha, India

E-mail: [jsundaray@gmail.com](mailto:jsundaray@gmail.com)

Phone: 9437166872, 91-674-2465421

Fax: 91-674-2465407

**Supp. Fig. 1.**Relative abundances (%) of bacterial species that showed ≥ 2% variation across all *L. catla* gut samples. Minimum and maximum relative abundances are indicated by orange and blue colour bars, respectively (a). Venn diagram showed the numbers of shared and unique OTUs between female and male *L. catla* gut samples. The % in the Venn diagram indicates the ratios of the sequences that are associated with the OTUs in total sequences in each group.

**Supp. Fig. 2.** Bar graph represents the differentially abundant bacterial taxa identified through LEfSe analysis between male and female *L. catla* gut communities. A bar on the positive side of x-axis indicates the taxa that had higher relative abundance in male *L. catla* gut than the female gut microbial communities. C: Class; O: Order; F: Family; G: Genus

**Supp. Fig. 3.** Pearson correlation analysis between serum hormone (11-KT, Estradiol, FSH, and LH) level and abundant microbe at phylum (a) and genus (b) level. The cells are colored based on the Pearson correlation coefficient (*r*) between the serum hormone level and microbial communities. Red colour represents negative correlation, blue represents positive correlation, and * represents that the correlation was significant at *p-*value < 0.05.


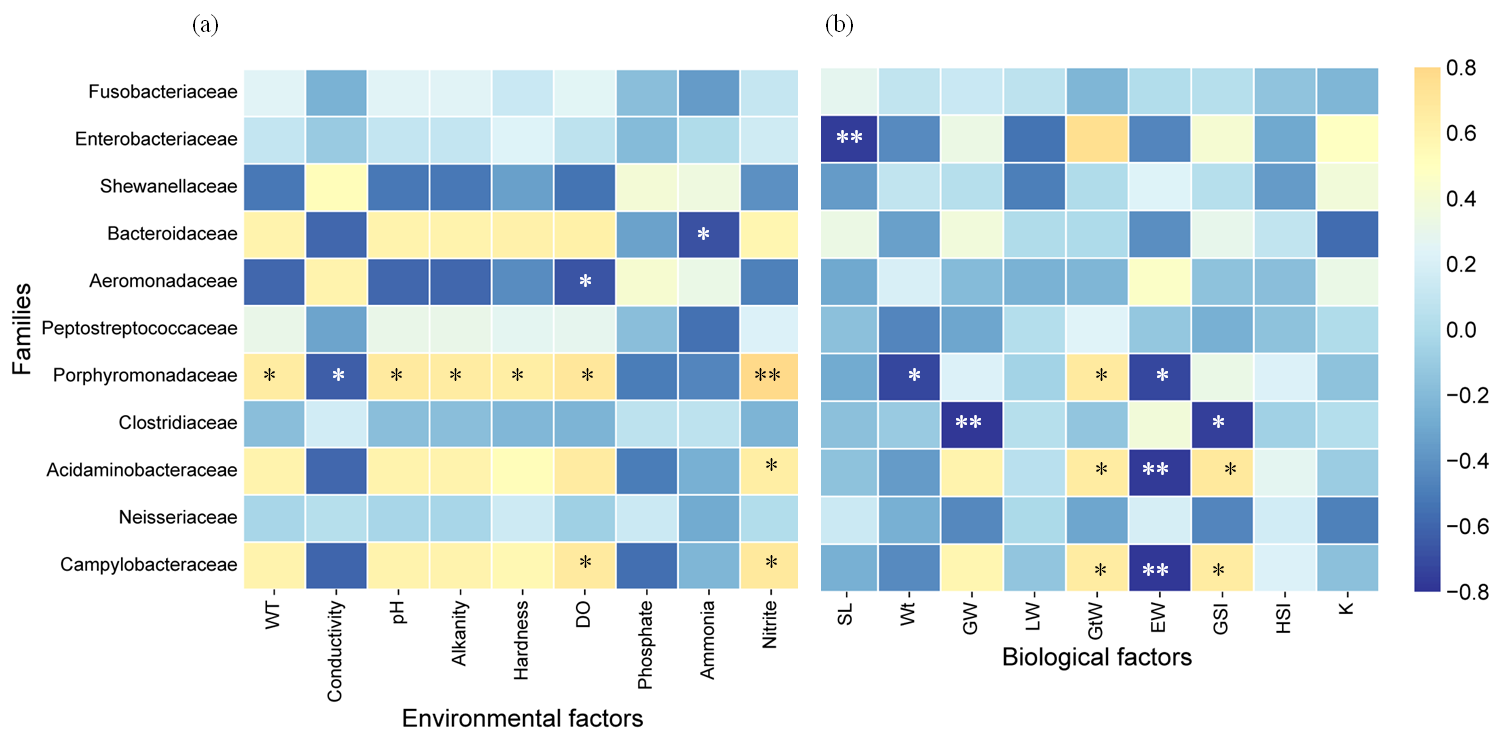


**Supp. Fig. 4.** Heatmap showing the correlation analysis between bacterial families and environmental factors (a) and bacterial families and biological factors (b). Color changes from blue to yellow represents the correlation coefficient changes from −0.8 to 0.8. Statistically significant (*p*-value < 0.05 or < 0.01) correlations are shown as single (*) or double asterisks (**), respectively. WT: water temperature; DO: dissolved oxygen; NO_2_^−^-N: nitrite-nitrogen, NH_4_^+^-N: ammonia-nitrogen, PO_4_^3−^-P: dissolved orthophosphate; W_t_: Body weight; SL: standard length; GW: gonad weight; LW: liver weight; GtW: gut weight; EW: Eviscerated weight; GSI, gonadosomatic index; HSI, hepatosomatic index; K, fatness

**Supp. Table 1.** Summary of raw and processed sequence data and quality of *L. catla* gut bacterial communities.

| **Sample Names** | **Raw_Reads** | **Processed_Reads** | **High Quality Data (%)** |
| --- | --- | --- | --- |
| CGF1 | 110804 | 98563 | 88.95 |
| CGF2 | 205166 | 183869 | 89.62 |
| CGF3 | 83108 | 75349 | 90.66 |
| CGF4 | 219805 | 196773 | 89.52 |
| CGF5 | 102556 | 92490 | 90.18 |
| CGM1 | 239487 | 211873 | 88.47 |
| CGM2 | 137426 | 124092 | 90.3 |
| CGM3 | 253102 | 227591 | 89.92 |
| CGM4 | 589064 | 530000 | 89.97 |
| CGM5 | 700169 | 643407 | 91.89 |
| **TOTAL** | **2640687** | **2384007** |  |

**Supp. Table 2.** Spearman’s correlation (*ρ*) between environmental factors and bacterial alpha-diversity (observed richness and Shannon). Correlation is significant at the 0.05 level (*) and 0.01 (**). WT: water temperature; DO: dissolved oxygen; NO_2_^−^-N: nitrite, NH_4_^+^-N: ammonia, PO_4_^3−^-P: phosphate; SL: standard length; W_t_: Body weight; GW: gonad weight; LW: liver weight; GtW: gut weight; EW: Eviscerated weight; GSI, gonadosomatic index; HSI, hepatosomatic index; K, fatness

| **Parameters** | **Sobs** | **Chao1** | **Shannon** | **Simpson** |
| --- | --- | --- | --- | --- |
| WT (◦C) | 0.453 | 0.522 | -0.104 | -0.035 |
| Conductivity | -0.453 | -0.522 | 0.104 | 0.035 |
| pH | 0.453 | 0.522 | -0.104 | -0.035 |
| Alkalinity (mg L^-1^) | 0.453 | 0.522 | -0.104 | -0.035 |
| Hardness (mg L^-1^) | 0.610 | .675^*^ | 0.033 | 0.125 |
| DO (mg L^-1^) | 0.409 | 0.469 | -0.007 | 0.054 |
| PO_4_^3^−-P (mg L^-1^) | -0.336 | -0.405 | 0.209 | 0.139 |
| NH_4_^+^-N (mg L^-1^) | -0.614 | -.679^*^ | -0.032 | -0.129 |
| NO_2_−-N (mg L^-1^) | 0.395 | 0.436 | -0.060 | -0.087 |
| SL | -0.420 | -0.383 | 0.086 | 0.006 |
| Wt | -.842^**^ | -.830^**^ | -0.164 | -0.273 |
| GW | -0.267 | -0.219 | -0.274 | -0.322 |
| LW | -0.304 | -0.249 | 0.170 | 0.073 |
| GtW | 0.505 | 0.486 | 0.109 | 0.219 |
| EW | -0.430 | -0.467 | -0.103 | -0.176 |
| GSI | -0.200 | -0.152 | -0.139 | -0.200 |
| HSI | -0.067 | -0.006 | 0.370 | 0.248 |
| K | -0.248 | -0.321 | -0.345 | -0.345 |

**Supp. Table 3.** List of top four keystone taxa (nodes with a high average degree, high closeness centrality, and low betweenness centrality values) in bacterial communities for co-occurrence network analysis.

| **Topological features** | | **Keystone taxa** | **Degree** | **Betweeness centrality** | **Closeness centrality** |
| --- | --- | --- | --- | --- | --- |
| **Sex-type** | **Male** | *Clostridium_perfringens* (*Clostridia*) | 10 | 19.62 | 0.70 |
|  |  | *Pseudomonas_stutzeri* (γ*-Proteobacteria*) | 10 | 19.62 | 0.70 |
|  |  | *Plesiomonas_shigelloides* (γ*-Proteobacteria*) | 8 | 16.02 | 0.64 |
|  |  | *Serratia_marcescens* (γ*-Proteobacteria*) | 8 | 7.43 | 0.62 |
|  | **Female** | *Clostridium_perfringens* (*Clostridia*) | 10 | 18.83 | 0.78 |
|  |  | *Pseudomonas_stutzeri* (γ-Proteobacteria) | 10 | 18.83 | 0.78 |
|  |  | *Plesiomonas_shigelloides* (γ*-Proteobacteria*) | 8 | 34.50 | 0.70 |
|  |  | *Serratia_marcescens* (γ*-Proteobacteria*) | 8 | 1.83 | 0.58 |
